# Synthesis of phosphonoacetate analogues of the second messenger adenosine 5′-diphosphate ribose (ADPR)†

**DOI:** 10.1039/c9ra09284f

**Published:** 2020-01-09

**Authors:** Ondřej Baszczyňski, Joanna M. Watt, Monika D. Rozewitz, Ralf Fliegert, Andreas H. Guse, Barry V. L. Potter

**Affiliations:** Medicinal Chemistry and Drug Discovery, Department of Pharmacology, University of Oxford Mansfield Road Oxford OX1 3QT UK; Wolfson Laboratory of Medicinal Chemistry, Department of Pharmacy and Pharmacology, University of Bath Bath BA2 7AY UK barry.potter@pharm.ox.ac.uk; The Calcium Signalling Group, Department of Biochemistry and Molecular Cell Biology, University Medical Center Hamburg-Eppendorf Martinistrasse 52 20246 Hamburg Germany

## Abstract

Adenosine 5′-diphosphate ribose (ADPR) is an intracellular signalling molecule generated from nicotinamide adenine dinucleotide (NAD^+^). Synthetic ADPR analogues can shed light on the mechanism of activation of ADPR targets and their downstream effects. Such chemical biology studies, however, are often challenging due to the negatively charged pyrophosphate that is also sensitive to cellular pyrophosphatases. Prior work on an initial ADPR target, the transient receptor potential cation channel TRPM2, showed complete pyrophosphate group replacement to be a step too far in maintaining biological activity. Thus, we designed ADPR analogues with just one of the negatively charged phosphate groups removed, by employing a phosphonoacetate linker. Synthesis of two novel phosphonoacetate ADPR analogues is described *via* tandem *N*,*N*′-dicyclohexylcarbodiimide coupling to phosphonoacetic acid. Neither analogue, however, showed significant agonist or antagonist activity towards TRPM2, underlining the importance of a complete pyrophosphate motif in activation of this particular receptor.

## Abstract 2

Pyrophosphate replacement using phosphonoacetate isosteres – tools to study biological targets of ADPR.

## Graphical abstract

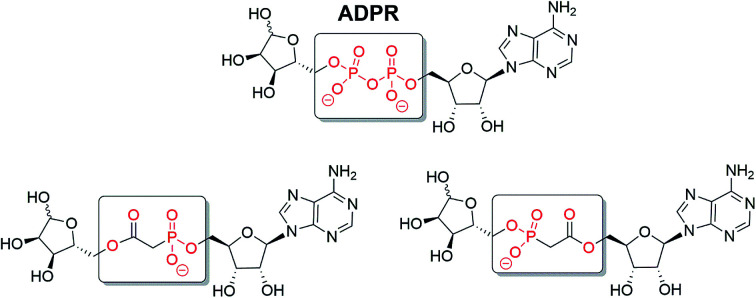

## Introduction

**Fig. 1:**
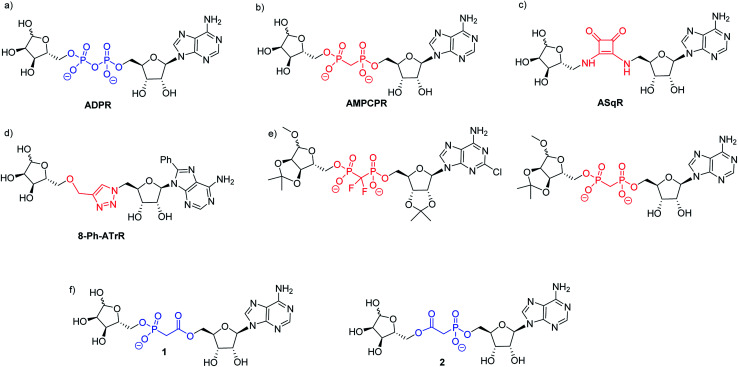
(a) ADPR; (b–e) ADPR analogues modified in the pyrophosphate motif and (f) the targets 1 and 2 of the present work.

The linear diphosphate adenosine 5′-diphosphate ribose (ADPR, Fig. 1a) is formed from nicotinamide adenine dinucleotide (NAD^+^) by two routes; either hydrolysis of the nicotinamide ribose by NAD^+^ glycohydrolases such as CD38 ^1^ or *via* the sequential action of poly-ADPR polymerase (PARP) and poly-ADPR glycohydrolase (PARG).^2^ PARP inhibitors are currently highly topical as drugs in oncology.^3^ ADPR's cyclic incarnation as cyclic ADP-ribose (cADPR) is a second messenger in cellular calcium signalling.^4,5^ cADPR is also hydrolysed to ADPR as a deactivation mechanism. ADPR targets include widespread macro domains^6^ and the non-selective cation channel, transient receptor potential cation channel, subfamily M, member 2 (TRPM2).^7,8^ They are associated with a diverse range of physiological processes that are still not yet fully understood. However, chemical biological studies of ADPR-mediated processes in whole cells are often hampered by ADPR's negative charges and lack of membrane permeability. Studies of these processes often require technically demanding and time-consuming patch clamp or microinjection experiments on single cells to deliver charged species physically through the cell membrane. Furthermore, ADPR is subject to hydrolysis of the pyrophosphate by enzymes (intracellular enzymes, ectoenzymes in the plasma membrane and enzymes present in biological fluids), including hydrolases of the NUDIX family. This means that ADPR itself is unlikely to be stable for any length of time in a biological setting, complicating the direct study of its effects. Thus, synthetic ADPR analogues that are both membrane permeant and stable in the biological environment would be valuable tools to unravel the downstream mechanisms and function of this ligand. Stable ADPR analogues may also be useful when clarification of the precise second messenger that is acting under physiological conditions has proved challenging.^9,10^

Small changes to the structure of ADPR have demonstrated profound effects on activity and, as a result, synthetic analogues closely based on the ADPR structure have been very informative.^11–13^ Analogues with modifications in the adenosine^11,13^ and terminal ribose^14–17^ motifs have been prepared *via* coupling of a modified 5′-*O*-phosphoryl adenosine or ribose to its 5′-*O*-phosphoryl counterpart using morpholidate, diphenylphosphonate or imidazolide methods to activate one phosphate towards nucleophilic attack and thus generate the pyrophosphate. Notwithstanding their utility for studying ADPR processes using single cell techniques such as patch clamping, further studies even with many of these designed analogues are limited by the negative charge and potential biological instability of the pyrophosphate moiety. Such pyrophosphate-containing ADPR analogues are thus not suitable for use in whole cell studies or for *in vivo* applications. The cell permeable peptide tat-M2NX has been shown to inhibit TRPM2 and reduce ischemic injury in male mice,^18^ suggesting that membrane permeable analogues closely related to ADPR in structure would find similar utility.

The negative charge of ADPR may be reduced by masking the charged phosphate groups. Indeed, ADPR that was acetylated and bioreversibly masked with a lipophilic group on the adenosine phosphate has been reported.^19^ However, pyrophosphates masked in this way can be more unstable towards hydrolysis by nucleophiles than their negatively charged counterparts, and this does not address the issue of degradation due to cellular pyrophosphatases. The use of phosphate bioisosteres in medicinal chemistry is an alternative method to improve cellular stability and potentially also improve membrane permeability.^20^ Previous studies using pyrophosphate replacement in ADPR have provided some new insights into ADPR binding. The methylene bis-phosphonate analogue of ADPR (α-β methylene ADPR, AMPCPR, Fig. 1b) contains a non-hydrolysable P–C–P pyrophosphate substitution that is stable towards pyrophosphatases and has therefore generated a number of crystal structures of ADPR targets bound to AMPCPR.^21^ Tóth *et al.* showed that AMPCPR still supports TRPM2 channel gating, albeit as a lower affinity partial agonist.^22^ AMPCPR has been used to uncouple the TRPM2 channel gating mechanism from its enzymatic activity by virtue of its pyrophosphatase resistant methylene bis-phosphonate linker.^22,23^ Two further ADPR analogues with similarly modified pyrophosphates (one methylene bisphosphate and one difluoromethylene bisphosphate, see Fig. 1e) that were also protected on some or all of the ADPR hydroxyl groups selectively inhibited TRPM2.^24^ These hydrolysis resistant analogues demonstrate that modification of the pyrophosphate can impart valuable attributes to the ADPR analogue. However, such methylene bis-phosphonate analogues still carry two negative charges at physiological pH, and thus are not membrane permeable.

Our previous highly ambitious complete replacements of the pyrophosphate group in ADPR (Fig. 1c and d), that are unlikely to be charged at physiological pH, unfortunately did not show TRPM2 agonist or antagonist activity.^11,13^ We therefore pursued a more conservative approach in this study, designing two ADPR analogues 1 and 2 with only one phosphorus centre replaced (Fig. 1f). We chose the isosteric phosphonoacetate group as a pyrophosphate mimic between the adenosine and the terminal ribose of ADPR, as this analogue would only carry a single negative charge at physiological pH. A phosphonoacetate motif should also be resistant to the intrinsic cellular pyrophosphatase activity of some ADPR targets as the P–C bond is more chemically stable than the pyrophosphate P–O bond.^25^ Therefore, such analogues could potentially be used to probe ADPR-mediated activities in isolation from pyrophosphatase activity. The first phosphonoacetate analogues of the 5-*O*-pyrophosphate in diphosphoinositol polyphosphates (PP-InsPs) were recognised and phosphorylated by the kinase domain of diphosphoinositol pentakisphosphate kinase 2 (PPIP5K2), showing that such substitution of a pyrophosphate did not prevent recognition of the analogue by its target enzyme and allowing downstream effects unrelated to dephosphorylation to be studied.^26^

We therefore report here the synthesis of phosphonoacetate ADPR analogues 1 and 2, studies on the stability of both analogues and their initial biological evaluation at TRPM2.

## Synthesis of ADPR analogues

**Scheme 1:**
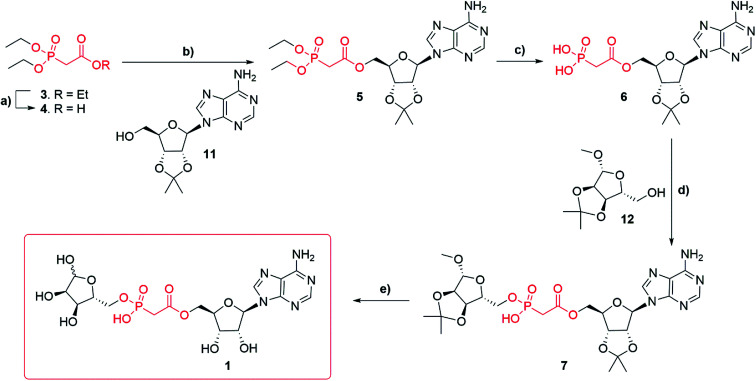
Synthesis of phosphonoacetate ADPR analogue 1. Reagents and conditions: (a) NaOH, water, EtOH, 20 °C (97%); (b) DCC, DMAP (cat.), THF, 20 °C (82%); (c) TMSBr, pyridine, 20 °C, 16 h (46%); (d) DCC, pyridine, 120 °C, 16 h (57%); (e) aqueous TFA 75%, 2 h, 20 °C (36%).

Synthesis of adenosine-5′-*O*-(2-phosphoryl) acetate ribose (Fig. 1f, 1) started with commercially available triethylphosphonoacetate 3 (Scheme 1). Selective deprotection of the carboxylic ester in the presence of the phosphate esters^27^ led to the corresponding carboxylic acid 4 (97%). Carboxylic acid 4 was esterified, by *N*,*N*′-dicyclohexylcarbodiimide (DCC) mediated coupling, with 2′,3′-*O*-isopropylidene adenosine 11 to obtain compound 5 (82%). Diethylphosphonate ester 5 was then trans-silylated by bromotrimethylsilane (TMSBr), followed by hydrolysis to obtain phosphonic acid 6 (best yield 46%). Selective removal of the phosphonate esters in the presence of the isopropylidene group was achieved by carrying out the reaction in pyridine, as the basic conditions buffer the HBr generated. Carrying out the same conversion with DCM as a solvent generated a complex mixture due to concomitant deprotection of the isopropylidene protecting group. Purification of the free phosphate 6 was carried out using normal phase flash chromatography on silica gel. Impurities were eluted first with an isocratic solvent system of ethyl acetate, methanol and water (7 : 2 : 1 v/v/v) containing 0.1% triethylamine. The desired product 6 was then eluted by switching to a second isocratic solvent system of isopropyl alcohol, water and aqueous ammonia (7 : 2 : 1 v/v/v) containing 0.1% triethylamine. First attempts to obtain compound 7*via* DCC mediated coupling to 1′-*O*-methyl-2′, 3′-*O*-isopropylidene-β-d-ribofuranoside 12 were unsuccessful. However, after a short optimization, we found that at elevated temperature (50–70 °C) compound 6 forms a mixture of anhydrides, including most likely adenosine phosphonoacetate anhydride^28^ which only reacts with 12 at temperatures above 100 °C. Thus, the higher temperature of 120 °C (compared to the DCC mediated reaction of 4 and 11 that occurred at rt) plays a crucial role in the conversion of 6 to 7. Finally, with the optimized conditions, the DCC mediated coupling of 6 with 1′-*O*-methyl-2′, 3′-*O*-isopropylidene-β-d-ribofuranoside 12 generated the desired ADPR analogue 7 (best yield 57%). Deprotection of 7 led to target compound 1, which was obtained after HPLC purification in 0.1 M TEAB buffer with a gradient of acetonitrile as the triethylammonium salt (36%). Until deprotection of analogue 7, all reactions are stereospecific. The final ADPR analogue 1 is a mixture of α and β anomers at the terminal ribose (ratio *α* : *β* = 2 : 3, as detailed in the Experimental section).

**Scheme 2:**
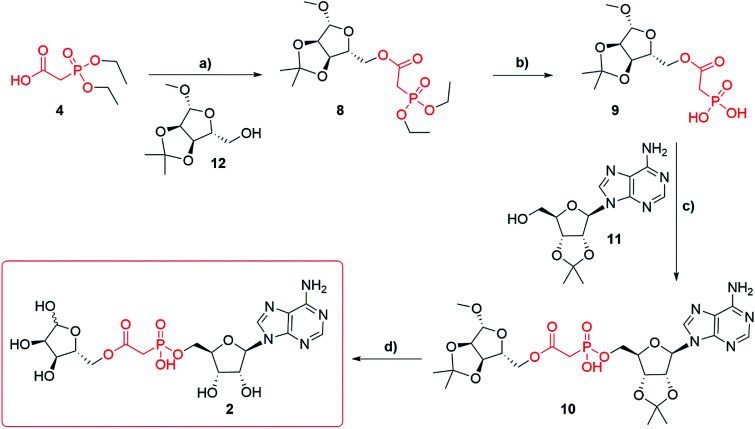
Synthesis of phosphonoacetate ADPR analogue 2. Reagents and conditions: (a) DCC, DMAP (cat.), THF, 20 °C (74%); (b) TMSBr, pyridine, 20 °C, 16 h (68%); (c) DCC, pyridine, 120 °C, 16 h (56%); (d) aqueous TFA 75%, 2 h, 20 °C (60%).

**Fig. 2:**
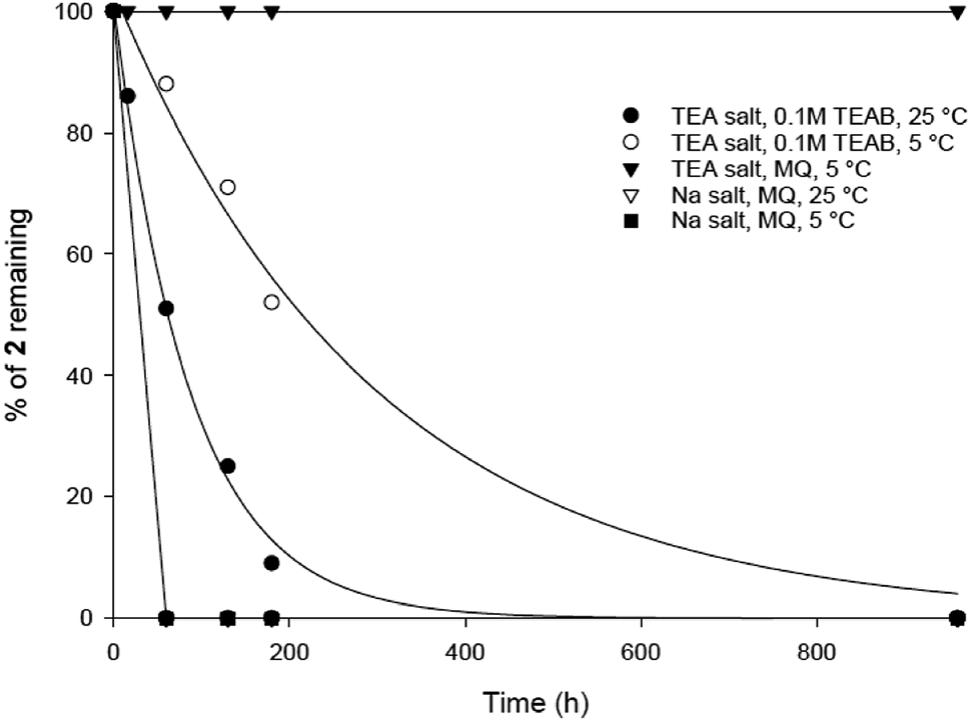
Stability of compound 2 under various aqueous conditions.

Synthesis of adenosine-5′-phosphonoacetyl ribose (Fig. 1f, 2) started from diethylphosphonoacetic acid 4 (Scheme 2), that was coupled to 1′-*O*-methyl-2′,3′-*O*-isopropylidene-β-d-ribofuranoside 12 using DCC, to give compound 8 (best yield 74%). Trans-silylation and hydrolysis of 8, followed by silica gel chromatography gave phosphonic acid 9 (68%) contaminated with trace impurities. Compound 9 was directly coupled to 2′,3′-*O*-isopropylidene adenosine 11*via* previously optimized DCC coupling at elevated temperature 120 °C to give compound 10 (56%). Deprotection of 10 led to the final compound 2, which was purified several times using HPLC in 0.1 M TEAB buffer with a gradient of acetonitrile as the triethylammonium salt (best yield 67%). Until deprotection of analogue 10, all reactions are stereospecific. The final ADPR analogue 2 is a mixture of α and β anomers at the terminal ribose (ratio *α* : *β* = 2 : 3, as detailed in the Experimental section).

## Stability of target phosphonoacetates 1 and 2

The stability of 1 and 2 in solution was monitored by analytical HPLC, comparing freshly made samples from the solid compounds 1 and 2, stored at −20 °C. Both final analogues were synthesised as the corresponding triethylammonium salts (1.0 × Et_3_N and 1.2 × Et_3_N equivalents respectively, calculated from ^1^H-NMR spectra). While phosphonoacetate 1 is stable in this form in MilliQ water, TEAB (0.1 M) or MeCN solutions, we were surprised to observe that phosphonoacetate 2, degrades slowly in TEAB (pH 8–9, 0.1 M TEAB, ESI, Fig. S1†). HPLC of 2 (*R*_t_ = 3.9 minutes) after 16 h in 0.1 M TEAB buffer showed an additional peak at *R*_t_ = 2.7 minutes. This decomposition product showed a characteristic adenosine UV absorption (*λ*_max_ = 259) and was analysed using mass spectrometry (ESI^+^ found [M − H]^−^ 388.0676, C_12_H_15_N_5_O_8_P requires 388.0664) suggesting loss of the terminal ribose to generate adenosine 5′-phosphonoacetate (ESI, Fig. S1†). We suspect it is most likely that 2 undergoes basic hydrolysis of the terminal ribose from the phosphonoacetate ester mediated by triethylamine from the TEAB buffer.

We then measured the stability of phosphonoacetate 2 under various aqueous conditions (Fig. 2). According to these findings, analogue 2 was stable in pure MilliQ water (pH 7) as the triethylammonium salt when stored in the fridge. Importantly, this should be sufficient for biological assays using the pure phosphonoacetate 2 when used immediately from a frozen sample. Increased pH 8–9 (in 0.1 M TEAB buffer) resulted in slow decomposition of 2 either at room temperature or when stored in the fridge. We prepared the sodium salt of analogue 2 in an attempt to overcome the possibility of triethylammonium counter ions generating a basic solution and thus causing the decomposition of 2. The triethylammonium salt of 2 was stirred with Chelex (Na^+^ form) resin, which was removed by filtration to generate the sodium salt of 2. However, we were surprised to find that the corresponding sodium salt of 2 was even more unstable and degraded completely either at room temperature or when stored in the fridge (Fig. 2).

It was rather unexpected that ADPR analogue 1 is stable under various aqueous conditions whereas ADPR analogue 2 is not. This led us to consider which feature of 2 might be responsible for the observed degradation. Our observations suggest that 2 is sensitive to basic hydrolysis of the phosphonoacetate ester in TEAB buffer but that, under the same conditions, the phosphonoacetate ester of 1 is stable. This suggests that the terminal ribose 5′′-*O*-ester may influence the stability in a way that is not observed with the adenosine ribose 5′-*O*-ester. The triethylamine base present in TEAB buffer may catalyse hydrolysis of the ester bond between the phosphonoacetate ester and the 5′′-*O*-terminal ribose. Indeed, we have previously observed that modifications to the terminal ribose can generate ADPR analogues that are particularly prone to instability.^14^ Analogue 1 is not susceptible to hydrolysis because the more stable phosphonate monoester is adjacent to the terminal ribose. Phosphate monoesters are stable under basic conditions due to the negative charge on the phosphonate oxygen preventing nucleophilic attack. Presumably, the 5′-*O*-ester in analogue 1 is more sterically hindered, due to its proximity to the less flexible adenosine, and thus is less susceptible to hydrolysis under mildly basic conditions. In future, synthesis of the attractive corresponding phosphonamide ADPR analogues in which the ester is replaced by an amide linker, may be one method to ensure stable analogues are generated, as exemplified in a recent synthesis of an inositol polyphosphate pyrophosphate analogue.^29^

## Biological evaluation of 1 and 2

Due to our stability studies, we were aware of the possibility of degradation of the unstable ester bond of compound 2. This would have been undesirable in our assay to determine the agonist and antagonist effects of 2. In order to prevent degradation, the compounds were reconstituted using MilliQ water (pH 7), which was shown to generate a stable solution of both compounds (Fig. 2). The assay was carried out at pH 7.2. Immediately before the assay for agonist or antagonist effects, the solution of the analogue to be tested was verified by HPLC. No degradation was detected during the entire sequence of testing, or even when the pH 7 solution of the analogue was stored at −80 °C for 1 year.

**Fig. 3:**
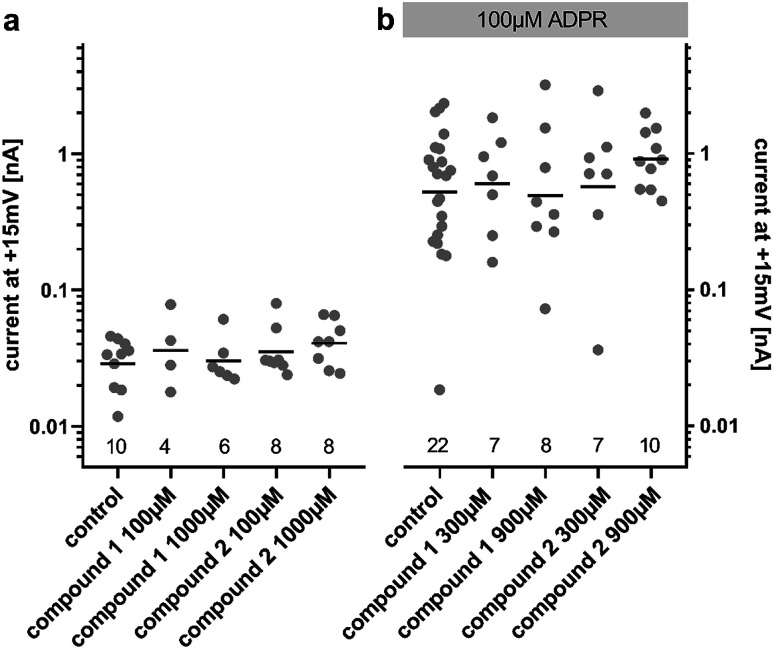
Effect of 1 and 2 on whole cell currents in hTRPM2 expressing HEK293 cells. Electrophysiological analysis of the impact of ADPR analogues on human TRPM2. (a) During whole cell patch clamp experiments using HEK293 cells with stable expression of the ion channel, the analogues were included in the pipette solution at 100 μmol L^−1^ or 1000 μmol L^−1^. The maximum outward current at +15 mV over a time of 450 s from individual cells is plotted at a logarithmic scale with a horizontal bar indicating the mean value. Neither compound did result in a significantly higher current than the buffer control. (b) To check for potential inhibition of TRPM2 currents from activation by ADPR, the compounds were added at 300 μmol L^−1^ or 900 μmol L^−1^ to a pipette solution already containing 100 μmol L^−1^ ADPR. Neither compound reduced the TRPM2 current significantly when compared to the control (pipette solution containing only ADPR). The numbers at the bottom indicate the number of cells analysed.

ADPR analogues 1 and 2 were tested for their agonist and antagonist activity on human TRPM2 (hTRPM2) using patch clamp experiments in HEK293 clones with stable expression of hTRPM2.^13^ Because the compound is infused into the cytoplasm of the cell *via* the patch-pipette, this method allows evaluation of the activity of the compounds independently of their membrane permeability. Following the results of our stability testing, phosphonoacetate 2 was stored as a lyophilized dry solid at −20 °C and made up in fresh MilliQ water immediately before testing to ensure that decomposition did not occur (*vide supra*). When applied at 100 μM or 1000 μM, neither 1 nor 2 had significant agonist effects at hTRPM2 (Fig. 3a). When applied with ADPR in the patch pipette (100 μM of ADPR with 300 μM or 900 μM 1 or 2), neither analogue showed significant antagonist effects on the ADPR-induced TRPM2 current (Fig. 3b). If activity was observed, we would have determined the membrane permeability of 1 and 2 by applying them extracellularly to whole cells, however this was not possible.

These results highlight the sensitivity of hTRPM2 channel activation to modifications of the ADPR molecular structure. It is possible that both phosphate motifs of the pyrophosphate bridge are required for binding and activation or inhibition of the channel, so that even a subtle change in this part of the molecule may lead to the complete loss of activity. The single atom substitution in the methylene bis-phosphonate analogue AMPCPR corresponded to a roughly 40 fold reduction in potency at hTRPM2 and AMPCPR is also only a partial hTRPM2 agonist.^22^ Alternatively the substitution of the pyrophosphate with the phosphonoacetate bioisostere may alter the presentation of other critical binding partners in the hTRPM2 binding pocket. Our molecular modelling suggests the phosphonoacetate linker theoretically seems to be a very good spatial mimic of the pyrophosphate unit, with sufficient flexibility to allow the adenosine and terminal ribose binding partners to position themselves in the same orientation as ADPR (ESI, Fig. S2†), but this may not reflect the reality once solvent effects and binding interactions are taken into account.

Cryo-EM structures of hTRPM2 have recently improved understanding of the zebrafish hTRPM2 apo and ADPR-bound states^30^ importantly for hTRPM2 also in the presence of bound ligands.^31,32^ However, only recently has their resolution become detailed enough to place ADPR in the binding pocket and has revealed, for the first time, ADPR binding sites in hTRPM2 that may act synergistically.^32^ The requirement for an ADPR mimic to bind to both sites, which possess very distinct shapes,^30^ may explain why very few synthetic ADPR analogues have activated the channel.^11,15^ Despite this recent advance, the structure activity relationship for hTRPM2 is not fully understood, and ADPR analogues remain essential for ligand-based drug design.

The crystal structure of the macro domain of thermophilic protein Af1521 bound to ADPR was solved in high resolution (1.5 Å, PDB 2BFQ).^6^ With the exception of the adenosine ribose, each ADPR structural feature interacts closely with the macro domain structure. The adenosine 5′-*O*-phosphate has two hydrogen bonds to the backbone N–H of valine 43 and glycine 143. The ribose 5′-*O*-phosphate has hydrogen bonds to the backbone N–H of serine 141 and tyrosine 145. Furthermore, the pyrophosphate is stabilized by the helix dipole. Thus, the demonstrated highly specific binding interactions of macro domains and apparent similar selectivity of hTRPM2 binding require very specific ADPR binding partners. Our initial results with hTRPM2 suggest that phosphonoacetate analogues 1 and 2 may not be able to fulfil all these demands, but 1 and 2 may potentially find applicability in other systems that bind ADPR.

## Conclusion

We have synthesised two phosphonoacetate analogues of ADPR, in which the pyrophosphate is substituted by a phosphonoacetate linker. These analogues retain all other ADPR features but would be predicted to carry only a single negative charge at physiological pH. Suitably protected ribose and adenosine building blocks were sequentially coupled to the phosphonoacetate core by carbodiimide-mediated coupling, followed by a global deprotection to give phosphonoacetates 1 and 2. Analogue 2, in which the phosphonoacetate ester is attached to the terminal ribose 5′′-hydroxyl, has unexpected instability in TEAB buffer. Future efforts would seek to avoid using ester links in such analogues, but likely employ amide linkages. Neither analogue showed any agonist activity at hTRPM2, nor antagonist effects on the ADPR-induced current at TRPM2, suggesting the full pyrophosphate motif is required for activity and adding to the growing picture of a highly specific binding interaction with many requisite ADPR features.

## Experimental section

### General

Reagents and solvents were purchased from commercial sources and used without further purification, unless described otherwise. Triethylamine was dried with potassium hydroxide, purified by distillation, and stored over potassium hydroxide. MilliQ quality water was used for all aqueous experiments and purifications. All reactions were performed under an inert atmosphere of argon unless otherwise stated. For NMR experiments; ^1^H, ^13^C, and ^31^P NMR spectra were collected using either a Varian Mercury 400 MHz or Bruker Avance III 500 MHz Spectrometer. All ^1^H and ^13^C NMR assignments are based on COSY, HSQC, HMBC, and DEPT experiments. Chemical shifts (*δ*) are reported in parts per million (ppm) and splitting patterns are abbreviated as follows: br, broad; s, singlet; d, doublet; t, triplet; m, multiplet *etc.* High resolution mass spectra (HRMS) were obtained on a Bruker Daltonics micrOTOF mass spectrometer with electrospray ionisation (ESI). Analytical HPLC was performed on a Waters 2695 Alliance module coupled to a Waters 2996 PDA Detector (210–350 nm) equipped with Hichrom Guard Column for HPLC and a Phenomenex Synergi column (4u, MAX-RP, 80 Å, 150 × 4.60 mm), eluted at 1 mL min^−1^ with a gradient of MeCN in 0.05 M TEAB. Semi-preparative HPLC was performed on a Waters 2525 pump with manual FlexInject using a Phenomenex Gemini column (5u, C18, 110 Å, 250 × 10.00 mm), eluted at 5 mL min^−1^.

#### 1-*O*-Methyl-2,3-*O*-isopropylidene-β-d-ribofuranoside (12)

Compound 12 was prepared according to the literature from d-ribose.^32^ Yield 63%. ^1^H NMR (400 MHz, CDCl_3_) *δ* 4.93 (s, 1H, H-1), 4.78 (d, *J* = 6.0 Hz, 1H, H-2), 4.54 (d, *J* = 6.0 Hz, 1H, H-3), 4.38 (bs, 1H, H-4), 3.66–3.55 (m, 2H, H-5_a,b_), 3.38 (s, 3H, OCH_3_), 3.19 (bs, 1H, OH), 1.44 (s, 3H, CH_3_), 1.27 (s, 3H, CH_3_).

#### Diethylphosphonoacetic acid (4)

Compound 4 was prepared from commercially available triethyl phosphonoacetate following the described procedure.^27^ Yield 97% of compound 4. ^1^H NMR (400 MHz, *d*_6_-DMSO) *δ* 12.6 (bs, 1H, OH), 4.09 (q, 4H, *J* = 7.2 Hz, **CH**_**2**_CH_3_), 3.02 (d, 2H, *J*_CH_2_–P_ = 21.2, **CH**_**2**_P), 1.29 (t, 6H, *J* = 7.2 Hz, CH_2_**CH**_**3**_). ^31^P NMR (400 MHz, *d*_6_-DMSO) *δ* 20.93 (s).

#### 5′-*O*-[(Diethoxyphosphoryl)acetate]-2′,3′-*O*-isopropylidene adenosine (5)

Diethylphosphonoacetic acid 4 (0.9 g, 4.59 mmol) and 2′,3′-*O*-isopropylidene adenosine (1 g, 3.25 mmol) were dissolved in THF (50 mL). *N*,*N*′-Dicyclohexylcarbodiimide (DCC, 1 g, 4.8 mmol) was added to the stirred solution in one portion, followed by catalytic amount of DMAP (20 mg, 0.16 mmol). The resulting mixture was stirred at room temperature overnight under argon atmosphere. The precipitate (*N*,*N*′-dicyclohexylurea) was filtered off and the solution was evaporated. The residue was dissolved in EtOAc, cooled to 5 °C, and filtered through a sintered glass filter. The solution was evaporated and the crude product was purified by Isco-Flash chromatography eluted with petroleum ether–EtOAc (6 : 4 v/v) and a gradient of MeOH (0–10%). This procedure afforded the title compound 5 as colourless glass (0.98 g, 62%). ^1^H NMR (400 MHz, *d*_6_-DMSO) *δ* 8.34 (s, H-8), 8.16 (s, H-2), 7.34 (s, 2H, NH_2_), 6.19 (d, 1H, *J* = 2.68 Hz, H-1′), 5.47 (dd, 1H, *J* = 6.0 Hz, *J* = 2.96 Hz, H-2′), 5.04 (dd, 1H, *J* = 6.4 Hz, *J* = 3.09 Hz, H-3′), 4.39–4.35 (m, 1H, H-4′), 4.31–4.27 
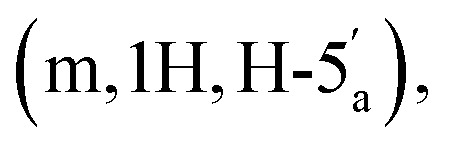
 4.23–4.18 
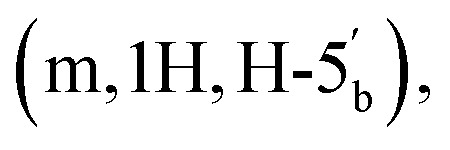
 4.04–3.95 (m, 4H, **CH**_**2**_CH_3_), 3.11 (d, 2H, *J*_H–P_ = 21.36 Hz, CH_2_P), 1.54 (s, 3H, C(**CH**_**3**_)_2_), 1.33 (s, 3H, C(**CH**_**3**_)_2_), 1.18 (m, 6H, *J* = 3.16 Hz, CH_2_**CH**_**3**_). ^31^P NMR (161 MHz, *d*_6_-DMSO) *δ* 19.64 (s). ^13^C NMR (100 MHz, *d*_6_-DMSO) *δ* 165.44 (COO), 156.14 (C-6), 152.75 (C-2), 148.81 (C-4), 139.83 (C-8), 119.08 (C-5), 113.49 (**C**(CH_3_)_2_), 89.00 (C-1′), 83.31 (C-4′), 83.08 (C-2′), 81.02 (C-3′), 64.56 (C-5′), 62.05 and 61.99 (**CH**_**2**_CH_3_), 33.22 (d, *J*_C–P_ = 130.57 Hz, C–P), 26.99 and 25.19 (C(**CH**_**3**_)_2_), 16.06 and 16.01 (CH_2_**CH**_**3**_). HRMS (ES^+^) calcd for C_19_H_29_N_5_O_8_P 486.1748, found [M + H]^+^ 486.1789.

#### 5′-*O*-Phosphonic acid-2′,3′-*O*-isopropylidene adenosine (6)

Bromotrimethylsilane (1.2 mL, 9.12 mmol) was added dropwise to a solution of 5′-*O*-[(diethoxyphosphoryl) acetate]-2′,3′-*O*-isopropylidene adenosine 5 (0.553 g, 1.14 mmol) in dry pyridine (6 mL) at 0 °C. The mixture was stirred at room temperature for 5 h and then another portion of bromotrimethylsilane (0.5 mL) was added dropwise. The mixture was stirred at room temperature and monitored *via* phosphorus NMR, ^31^P NMR (161 MHz, *d*_6_-DMSO) *δ* 8.14. After 16 h, the solvent was evaporated and the residue was co-evaporated with aqueous ammonia. The crude material was purified by silica gel chromatography. Impurities were eluted with EtOAc–MeOH–H_2_O (7 : 2 : 1 v/v/v + 0.1% Et_3_N) and the product was eluted using iPrOH–H_2_O–aq. ammonia (7 : 2 : 1 v/v/v + 0.1% Et_3_N). This procedure afforded the title compound 6 as a white foam, as the mono-triethylammonium salt (179 mg, 30%). ^1^H NMR (400 MHz, *d*_6_-DMSO) *δ* 8.59 (s, 1H, H-8), 8.16 (s, 1H, H-2), 7.33 (s, 2H, NH_2_), 6.13 (d, 1H, *J* = 3.16 Hz, H-1′), 5.54 (dd, 1H, *J* = 6.4 Hz, *J* = 3.2 Hz, H-2′), 5.10 (dd, 1H, *J* = 6.0 Hz, *J* = 3.0 Hz, H-3′), 4.39 (m, 1H, H-4′), 4.22 
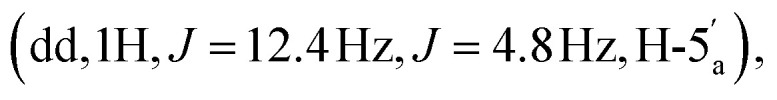
 4.06 
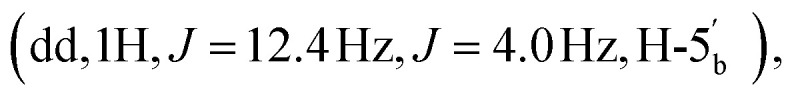
 2.89 (q, 6H, *J* = 7.25, N**CH**_**2**_CH_3_), 2.53 (dq, 2H, *J*_H–P_ = 22.4 Hz, *J*_gem_ = 8.0 Hz, CH_2_P), 1.53 (s, 3H, C(**CH**_**3**_)_2_), 1.18 (s, 3H, C(**CH**_**3**_)_2_), 1.11 (t, 6H, *J* = 7.26 Hz, NCH_2_**CH**_**3**_). ^31^P NMR (161 MHz, *d*_6_-DMSO) *δ* 8.14 (s). ^13^C NMR (100 MHz, *d*_6_-DMSO) *δ* 176.66 (COO) HMBC, 156.06 (C-6), 152.76 (C-2), 149.13 (C-4), 139.84 (C-8), 118.79 (C-5), 113.27 (**C**(CH_3_)_2_), 88.69 (C-1′), 83.40 (C-4′), 83.16 (C-2′), 81.01 (C-3′), 63.52 (C-5′), 45.05 (N**CH**_**2**_CH_3_), (d, 38.52 and 38.02, very low intensity, CH_2_P signal partially hidden by the DMSO signal, estimated from HSQC), 27.02 and 25.20 (C(**CH**_**3**_)_2_), 8.70 (NCH_2_**CH**_**3**_). HRMS (ES^−^) calcd for C_15_H_19_N_5_O_8_P 428.0977, found [M − H]^−^ 428.0985.

#### 2′,3′-*O*-Isopropylidene adenosine-5′-*O*-(2-phosphoryl)acetate-1′′-*O*-methyl-2′′,3′′-*O*-isopropylidene-β-d-ribofuranoside (7)

5′-*O*-Phosphonic acid-2′,3′-*O*-isopropylidene adenosine 6 (28 mg, 0.053 mmol) and 1-*O*-methyl-2,3-*O*-isopropylidene-β-d-ribofuranoside 12 (20 mg, 0.098 mmol) were dissolved in anhydrous pyridine (1 mL). *N*,*N*′-dicyclohexylcarbodiimide (20 mg, 0.097 mmol) was added in one portion to the stirred mixture and the mixture was heated at 120 °C for 16 h. The reaction mixture was evaporated to dryness and the compound was purified by silica-gel chromatography CH_2_Cl_2_–MeOH-0.1% Et_3_N (10–40%) to afford the title compound 7 as a white solid (18.6 mg, 54%, ⅛ Et_3_N salt). ^1^H NMR (400 MHz, *d*_6_-DMSO) *δ* 8.69 (s, 1H, H-8), 8.16 (s, 1H, H-2), 7.34 (bs, 2H, NH_2_), 6.13 (d, 1H, *J* = 3.0 Hz, H-1′), 5.56 (q, 1H, *J* = 2.99 Hz, H-2′), 5.07 (dd, 1H, *J* = 6.4 Hz, *J* = 2.93 Hz, H-3′), 4.86 (s, 1H, H-1′′), 4.75 (d, 1H, *J* = 5.96 Hz, H-2′′), 4.52 (d, 1H, *J* = 5.96 Hz, H-3′′), 4.42–4.39 (m, 1H, H-4′), 4.26–4.22 
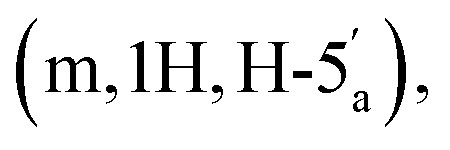
 4.15–4.11 (m, 1H, H-4′′), 4.06–4.02 
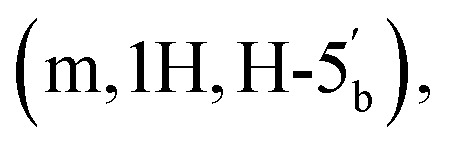
 3.66–3.57 
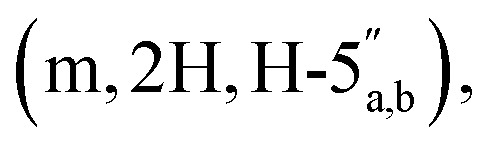
 3.53 (q, 0.7H, *J* = 6.32 Hz, N**CH**_**2**_CH_3_), 3.19 (s, 3H, CH_3_O), 2.60–2.47 (m, 2H, CH_2_P), 1.53 and 1.33 (2 × s, 2 × 3H, C(**CH**_**3**_)_2_-Adn), 1.36 and 1.23 (2 × s, 2 × 3H, C(**CH**_**3**_)_2_-Rib). ^31^P NMR (161 MHz, *d*_6_-DMSO) *δ* 7.60 (s). ^13^C NMR (100 MHz, *d*_6_-DMSO) *δ* 169.60 (COO) HMBC, 156.04 (C-6), 152.76 (C-2), 149.13 (C-4), 140.15 (C-8), 118.75 (C-5), 113.23 (Adn **C**(CH_3_)_2_), 111.22 (Rib **C**(CH_3_)_2_), 108.45 (C-1′′), 88.75 (C-1′), 85.18 (C-4′′), 84.42 (C-3′′), 83.52 (C-4′), 83.30 (C-2′), 81.46 (C-2′′), 81.04 (C-3′), 64.00 (C-5′′), 63.50 (C-5′), 54.15 (CH_3_O), 36.32 (d, *J* = 112.0 Hz, CH_2_P a and b, HMBC), 27.03 and 25.21 (C(**CH**_**3**_)_2_-Adn), 26.22 and 24.61 (C(**CH**_**3**_)_2_-Rib). HRMS (ES^−^) calcd for C_24_H_33_N_5_O_12_P 614.1869, found [M − H]^−^ 614.1885. Analytical HPLC, *R*_t_ = 8.62 min.

#### Adenosine-5′-*O*-(2-phosphoryl)acetate ribose (1)

2′,3′-*O*-Isopropylidene adenosine-5′-*O*-(2-phosphoryl) acetate-1′′-*O*-methyl-2′′,3′′-*O*-isopropylidene-β-d-ribofuranoside 7 (10.7 mg, 0.0165 mmol) was dissolved in 75% aqueous CF_3_COOH (8 mL) and the mixture was stirred at room temperature for 2 h. The reaction mixture was quickly evaporated at 30 °C to dryness and the residue was co-evaporated (3×) with Milli-Q water. The crude product was dissolved in triethylammonium bicarbonate buffer (TEAB, 2 mL, 0.1 M) and purified by semi-preparative, reverse-phase HPLC using a gradient of 0.1 M TEAB-acetonitrile (95 : 5 → 35/65 v/v). The title compound 1 was afforded as colourless glass, as a mixture of α and β anomers in the ratio *α* : *β* = 2 : 3, (3.1 mg, 36%, ⅓ Et_3_N salt). DOSY ^1^H NMR (water suppression, 500 MHz, D_2_O) *δ* 8.32 (s, 1H, H-8), 8.18 (s, 1H, H-2), 6.03 (d, 1H, *J* = 4.5 Hz, H-1′), 5.16 (d, 0.4H, *J* = 4.0 Hz, H-1′′α), 5.10 (d, 0.6H, *J* = 2.5 Hz, H-1′′β), 4.77 (t, 1H, obscured by D_2_O peak, *J* = 2.5 Hz, H-2′), 4.45 (t, 1H, *J* = 5.0 Hz, H-3′), 4.41–4.32 (m, 3H, H-4′, 5′), 4.12 (t, 0.6H, *J* = 5.0 Hz, H-3′′β), 4.07–4.04 (m, 0.4H, H-4′′α), 4.01–3.75 
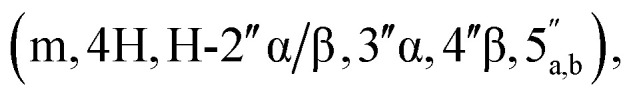
 3.12 (q, 2H, *J* = 7.0 Hz, N**CH**_**2**_CH_3_), 2.81 and 2.79 (2 × dd, 2H, *J*_H–P_ = 15.5 and 16 Hz, *J*_gem_ = 5.0 and 4.5 Hz, CH_2_P α, β), 1.20 (t, 3H, *J* = 7.0 Hz, NCH_2_**CH**_**3**_). ^31^P NMR (203 MHz, D_2_O) *δ* 14.40 (s), 14.30 (s). ^13^C NMR (126 MHz, D_2_O) *δ* 169.70 (COO) HMBC, 155.53 (C-6), 152.73 (C-2), 149.02 (C-4), 139.99 (C-8), 118.87 (C-5), 101.05 (C-1′′β), 96.28 (C-1′′α), 87.58 (C-1′), 81.85 and 81.16 (C-4′, 4′′α and β), 75.06 (C-2′′β), 73.44 (C-2′), 70.65, 70.44, 70.00, 69.85 (C-2′′α, 3′′α/β, 3′), 65.31 (d, *J* = 4.5 Hz, C-5′′), 64.00 (d, *J* = 5.5 Hz, C-5′), 46.65 (N**CH**_**2**_CH_3_), 35.03 and 34.34 (d, *J*_C–P_ = 121.4 Hz, CH_2_P), 35.27 and 34.29 (d, *J*_C–P_ = 122.1 Hz, CH_2_P), 8.19 (NCH_2_**CH**_**3**_). HRMS (ES^−^) calcd for C_17_H_23_N_5_O_12_P 520.1086, found [M − H]^−^ 520.1097. Analytical HPLC: *R*_t_ = 4.14 min.

#### 5-*O*-[(Diethoxyphosphoryl)acetate]-1-*O*-methyl-2, 3-*O*-isopropylidene-β-d-ribofuranoside (8)

1-*O*-Methyl-2,3-*O*-isopropylidene-β-d-ribofuranoside 12 (100 mg, 0.49 mmol) and diethylphosphonoacetic acid 4 (96 mg, 0.49 mmol) were dissolved in dry THF (5 mL). *N*,*N*′-Dicyclohexylcarbodiimide (142 mg, 0.68 mmol) was added to the stirred solution in one portion followed by catalytic amount of DMAP (2 mg, 0.016 mmol). The resulting mixture was stirred at room temperature for 16 h under argon. The precipitate (*N*,*N*′-dicyclohexylurea) was filtered off and the solution was evaporated to dryness. The residue was dissolved in EtOAc, cooled to 5 °C, and filtered through a sintered glass filter. The solution was evaporated to dryness and the crude product was purified by Isco-Flash chromatography eluting with petroleum ether–EtOAc (1 : 0 → 0 : 1 v/v) to afford the title compound 8 as a colourless glass (112 mg, 60%). ^1^H NMR (400 MHz, CDCl_3_) *δ* 4.96 (s, 1H, H-1), 4.67 (d, 1H, *J* = 6.0 Hz, H-2), 4.59 (d, 1H, *J* = 6.0 Hz, H-3), 4.35 (t, 1H, *J* = 7.2 Hz, H-4), 4.21–4.13 (m, 6H, H-5, **CH**_**2**_CH_3_), 3.31 (s, 3H, OCH_3_), 2.98 (d, 2H, *J* = 21.6, **CH**_**2**_P), 1.46 (s, 3H, C(**CH**_**3**_)_2_), 1.34 (t, 6H, *J* = 7.2 Hz, CH_2_**CH**_**3**_), 1.30 (s, 3H, C(**CH**_**3**_)_2_). ^31^P NMR (161 MHz, CDCl_3_) *δ* 19.22. ^13^C NMR (100 MHz, CDCl_3_) *δ* 165.72 (COO), 112.90 (**C-**(CH_3_)_2_), 109.79 (C-1), 85.50 (C-3), 84.29 (C-4), 82.15 (C-2), 65.88 (C-5), 63.19 (d, *J*_C–P_ = 6.06 Hz, **CH**_**2**_CH_3_), 55.34 (OCH_3_), 34.67 (d, *J*_C–P_ = 133.79 Hz, CH_2_P), 26.73 (CH_3_), 25.29 (CH_3_), 16.70 (CH_2_**CH**_**3**_). HRMS (ES^+^) calcd for C_15_H_28_O_9_P 383.1465, found [M + H]^+^ 383.1474.

#### 5-*O*-Phosphonic acid-1-*O*-methyl-2,3-*O*-isopropylidene-β-d-ribofuranoside (9)

Bromotrimethylsilane (1.33 mL, 10.1 mmol) was added dropwise to the solution of 5-*O*-[(diethoxyphosphoryl) acetate]-1-*O*-methyl-2,3-*O*-isopropylidene-β-d-ribofuranoside 8 (0.642 g, 1.68 mmol) and triethylamine (4.18 mL, 30.25 mmol) in dry dichloromethane (20 mL) at 0 °C. The mixture was stirred at room temperature for 16 h and monitored *via* phosphorus NMR, ^31^P NMR (161 MHz, *d*_6_-DMSO) *δ* 8.36. The solution was evaporated to dryness and the residue was co-evaporated with aqueous ammonia. The crude material was purified by silica gel chromatography. Impurities were eluted with EtOAc–MeOH–H_2_O (7 : 2 : 1 v/v/v + 0.1% Et_3_N) and the product was eluted using iPrOH–H_2_O–aq. ammonia (7 : 2 : 1 v/v/v + 0.1% Et_3_N) to afford phosphonic acid 9 (603 mg, 68%, mono-triethylammonium salt) which was used for the next reaction without any further purification. ^1^H NMR (400 MHz, *d*_6_-DMSO) *δ* 4.91 (s, 1H, H-1), 4.75 (d, 1H, *J* = 7.5 Hz, H-2), 4.57 (d, 1H, *J* = 7.5 Hz, H-3), 4.22 (t, 1H, *J* = 9.0 Hz, H-4), 3.98–3.86 (m, 2H, H-5), 3.22 (s, 3H, MeO), 2.78 (q, 2H, *J* = 9.0 Hz, CH_2_P), 1.37 (s, 3H, C(**CH**_**3**_)_2_), 1.25 (s, 3H, C(**CH**_**3**_)_2_). ^31^P NMR (161 MHz, *d*_6_-DMSO) *δ* 8.36 (s). ^13^C NMR (400 MHz, *d*_6_-DMSO) *δ* 168.51 (d, *J* = 6.5 Hz, COO), 111.44 (**C**(CH_3_)_2_), 108.73 (C-1), 84.44 (C-3), 83.37 (C-4), 81.23 (C-2), 64.03 (C-5), 54.23 (MeO), 44.82 (**CH**_**2**_P), 26.20 and 24.63 (2 × C(**CH**_**3**_)_2_). HRMS (ES^−^) calcd for C_11_H_18_O_9_P 325.0694, found [M − H]^−^ 325.0702.

#### 2′,3′-*O*-Isopropylidene adenosine-5′-*O*-phosphonoacetyl-1′′-*O*-methyl-2′′,3′′-*O*-isopropylidene-β-d-ribofuranoside (10)

5-*O*-Phosphonic acid-1-*O*-methyl-2,3-*O*-isopropylidene-β-d-ribofuranoside 9 (65 mg, 0.123 mmol) and 2′,3′-*O*-isopropylidene adenosine 11 (57 mg, 0.185 mmol) were dissolved in anhydrous pyridine (2 mL). *N*,*N*′-Dicyclohexylcarbodiimide (51 mg, 0.246 mmol) was added in one portion to the stirred mixture and the mixture heated to 120 °C for 16 h. The reaction mixture was evaporated to dryness and the compound was purified by silica-gel chromatography eluted with CH_2_Cl_2_–MeOH (9 : 1 → 6 : 4 v/v + 0.1% Et_3_N) to afford the title compound 10 as a white solid (52 mg, 56%). ^1^H NMR (500 MHz, D_2_O) *δ* 8.41 (s, 1H, H-8), 8.24 (s, 1H, H-2), 6.27 (d, 1H, *J* = 3.0 Hz, H-1′), 5.43–5.41 (m, 1H, H-2′), 5.20 (dd, 1H, *J* = 5.0 Hz, *J* = 2.0 Hz, H-3′), 4.93 (s, 1H, H-1′′), 4.71–4.66 (m, 1H, H-4′), 4.58 (d, *J* = 6.0 Hz, H-2′′), 4.43 (d, 1H, *J* = 6.0 Hz, H-3′′), 4.18–4.12 (m, 3H, H-4′′, 5′), 4.00–32.96 (m, 1H, H-5′′a), 3.86–3.81 (m, 1H, H-5′′b), 3.23 (s, 3H, CH_3_O), 2.84 (dq, 2H, *J*_H–P_ = 20.5 Hz, *J* = 13.5 Hz, *J*_gem_ = 5.0 Hz, CH_2_P), 1.68 (s, 3H, C(**CH**_**3**_)_2_-Adn), 1.40 (s, 3H, C(**CH**_**3**_)_2_-Adn), 1.46 (s, 3H, C(**CH**_**3**_)_2_-Rib), 1.27 (s, 3H, C(**CH**_**3**_)_2_-Rib). ^31^P NMR (161 MHz, D_2_O) *δ* 14.11 (s). ^13^C NMR (126 MHz, D_2_O) *δ* 169.17 (d, *J* = 6.8 Hz, COO), 155.56 (C-6), 152.79 (C-2), 148.78 (C-4), 139.86 (C-8), 118.55 (C-5), 114.76 (**C**(CH_3_)_2_-Adn), 112.96 (**C**(CH_3_)_2_-Rib), 108.69 (C-1′′), 90.16 (C-1′), 84.90 (d, *J* = 8.78 Hz, C-4′), 84.06 (C-3′′), 83.96 (C-2′), 83.40 (C-4′′), 81.46 (C-3′), 80.94 (C-2′′), 65.17 (C-5′′), 64.80 (d, *J* = 5.41 Hz, C-5′), 54.69 (CH_3_O), 34.89 and 33.93 (d, *J*_C–P_ = 120.5 Hz, CH_2_P), 26.14 and 24.35 (2 × C(**CH**_**3**_)_2_-Adn), 25.06 and 23.41 (2 × C(**CH**_**3**_)_2_-Rib); HRMS (ES^−^) calcd for C_24_H_33_N_5_O_12_P 614.1869, found [M − H]^−^ 614.1889.

#### Adenosine-5′-phosphonoacetyl-ribose (2)

2′,3′-*O*-Isopropylidene adenosine-5′-*O*-phosphonoacetyl-1′′-*O*-methyl-2′′,3′′-*O*-isopropylidene-β-d-ribofuranoside 10 (15.0 mg, 0.024 mmol) was dissolved in 75% aqueous CF_3_COOH (8 mL) and the mixture was stirred at room temperature for 2 h. The reaction mixture was quickly evaporated at 30 °C to dryness and the residue was co-evaporated with water (3×). The crude product was dissolved in triethylammonium bicarbonate buffer (TEAB, 2 mL, 0.1 M) and purified by semi-preparative, reverse-phase HPLC eluting with a gradient of 0.1 M TEAB-acetonitrile (95 : 5 → 35 : 65 v/v) to afford the title compound 2 as a colourless glass, as a mixture of α and β anomers α/β = 2/3 (7.6 mg, 60%, ⅓ Et_3_N salt). DOSY ^1^H NMR (water suppression, 500 MHz, D_2_O) *δ* 8.37 (s, 1H, H-8), 8.15 (s, 1H, H-2), 6.04 (d, 1H, *J* = 5.5 Hz, H-1′), 5.21–5.19 (m, 0.4H, H-1′′α), 5.11 (d, 0.6H, *J* = 1.5 Hz, H-1′′β), 4.67 (t, 1H, *J* = 5.0 Hz, H-2′), 4.44–4.40 (m, 1H, H-3′), 4.31–4.27 (m, 1H, H-4′), 4.24 
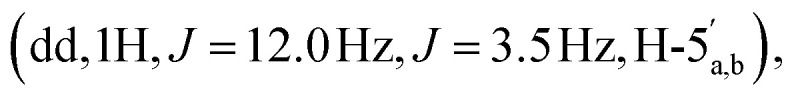
 4.71–3.96 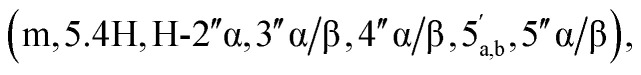 3.90–3.88 (m, 0.6H, H-2′′β), 3.11 (q, 2H, *J* = 7.0 Hz, N**CH**_**2**_CH_3_), 2.87–2.79 (m, 2H, CH_2_P), 1.19 (t, 3H, *J* = 7.5, NCH_2_**CH**_**3**_). ^31^P NMR (203 MHz, D_2_O) *δ* 14.61 (s), 14.50 (s). ^13^C NMR (126 MHz, D_2_O) *δ* 169.72 (COO), 155.52 (C-6), 152.77 (C-2), 149.1 (C-4), 139.74 (C-8), 118.69 (C-5), 101.18 (C-1′′β), 96.34 (C-1′′α), 87.02 (C-1′), 83.76 (C-4′), 79.83 and 79.44 (C-3′′α/β, 4′′α/β), 74.93 (C-2′′β), 74.20 (C-2′), 70.50 and 70.28 and 70.10 (C-3′′α/β, 2′′α, 3′), 65.30 
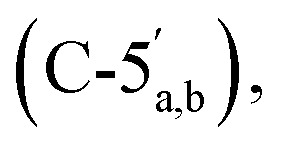
 64.58 and 63.84 (C-5′′α/β), 46.63 (N**CH**_**2**_CH_3_), 35.14 and 34.18 (d, *J*_C–P_ = 121.1 Hz, CH_2_P a and b), 8.18 (NCH_2_**CH**_**3**_). HRMS (ES^−^) calcd for C_17_H_23_N_5_O_12_P 520.1086, found [M − H]^−^ 520.1086. Analytical HPLC: RT = 3.68 min.

### Pharmacology

#### Cell culture

HEK293 cells were kept in DMEM medium (4.5 g L^−1^ glucose, Glutamax-I) supplemented with 10% FBS and penicillin (100 U mL^−1^) and streptomycin (100 μg mL^−1^) at 37 °C and 5% CO_2_. The medium for the stable cell lines additionally contained 400 μg mL^−1^ G418 sulfate. The parental HEK293 cells used to generate the cell line with stable expression of human TRPM2 were provided by Prof. Dr Manfred Jücker (Institute of Biochemistry and Signal Transduction, University Medical Centre Hamburg-Eppendorf). The cells were authenticated by short tandem repeat profiling (DSMZ service for the authentication of cell lines, https://www.dsmz.de/services/human-and-animal-cell-lines/authentication-of-human-cell-lines) and are being tested regularly regarding mycoplasma contamination using an enzymatic assay (MycoAlert mycoplasma detection kit; Lonza).

#### Generation of HEK293 cells with stable expression of TRPM2

HEK293 cells with stable expression of human TRPM2 and EGFP were generated as described before.^13^ In brief the cells were transfected with pIRES2-EGFP-TRPM2 (containing the coding sequence for the full-length of human TRPM2 under a CMV promoter followed by an internal ribosome entry site (IRES) and the coding sequence for EGFP). Cells that remained untransfected were killed by addition of G418 sulfate to the medium. Surviving cells were subjected to limiting dilution cloning. The derived clones were then tested for TRPM2 expression by Ca^2+^ measurement and patch-clamp analysis.

#### Whole cell patch-clamp recordings

Whole cell patch-clamp recordings were done as described before.^13^ In brief, HEK cells with stable expression of human TRPM2 were seeded to 35 mm dishes and kept as described above. 24 h after seeding the culture medium was removed and replaced by an extracellular solution (in mmol L^−1^: 140 NMDG, 5 KCl, 3.3 MgCl_2_, 1 CaCl_2_, 5 d-glucose, 10 HEPES, pH 7.4 with HCl). All recordings were done at room temperature. Patch pipettes were prepared from borosilicate glass tubing 1.05 × 1.50 × 80 mm, Science Products (Hofheim, Germany) using a Sutter P-97 horizontal puller resulting in a pipette resistance of 1.5 MΩ to 3.5 MΩ. An EPC-10 amplifier was used in conjunction with PatchMaster (v2x32, HEKA Elektronik, Lambrecht/Germany) to record the data. After break-in series resistance was compensated to 70% and the holding potential set to −50 mV. A total of 90 voltage ramps from −85 mV to +20 mV over 140 ms were applied every 5 s to follow activation of TRPM2 activation by ADPR or ADPR analogues added to the pipette solution (in mmol L^−1^: 120 KCl, 8 NaCl, 1 MgCl_2_, 10 HEPES, 10 EGTA, 5.6 CaCl_2_, pH 7.2 with KOH, 200 nmol L^−1^ free Ca^2+^ as calculated with MaxChelator (https://somapp.ucdmc.ucdavis.edu/pharmacology/bers/maxchelator/CaEGTA-NIST.htm)). The peak current at +15 mV during the course of the recording was taken for further quantitative analysis of TRPM2 activity.

#### Statistics

For statistical evaluation of patch-clamp data GraphPad Prism (version 7.04, GraphPad Software Inc.) was used. Currents from the patch-clamp recordings showed a positively skewed distribution and were normalized by log-transformation. The normalized data were tested using one-way-ANOVA with *post-hoc* testing against the appropriate control using Bonferroni correction for multiple testing (*α* = 0.05).

## Author contributions

J. M. W. and B. V. L. P. designed the study. O. B. and J. M. W. equally contributed and O. B. synthesized the ADPR analogues supervised by J. M. W.; M. D. R. carried out patch-clamp experiments with R. F. and A. H. G.; O. B., J. M. W. and B. V. L. P. wrote the manuscript with input from all authors.

## Conflicts of interest

There are no conflicts to declare.

## Supplementary Material

RA-010-C9RA09284F-s001
