# Supplementary material for: Synthesis of phosphonoacetate analogues of the second messenger adenosine 5′-diphosphate ribose (ADPR)
Source: RSC Adv. 2020 Jan 9;10(3):1776–85. doi: 10.1039/c9ra09284f (PMC6957348; doi:10.1039/c9ra09284f)
Supplement: RA-010-C9RA09284F-s001 [file RA-010-C9RA09284F-s001.pdf]

Electronic Supplementary Material (ESI) for RSC Advances.
This journal is © The Royal Society of Chemistry 2019

Supplementary Information

Synthesis of phosphonoacetate analogues of the second messenger adenosine 5'-diphosphate ribose (ADPR)

Ondřej Baszczyňski[2‖], Joanna M. Watt[1,2], Monika D. Rozewitz[3], Ralf Fliegert[3], Andreas H. Guse[3], and Barry V. L. Potter[1,2*]

[1] Medicinal Chemistry, Department of Pharmacology, University of Oxford, Mansfield Road, Oxford, OX1 3QT, UK

[2]Wolfson Laboratory of Medicinal Chemistry, Department of Pharmacy and Pharmacology, University of Bath, Bath, BA2 7AY, UK

[3]The Calcium Signalling Group, Department of Biochemistry and Molecular Cell Biology, University Medical Center Hamburg-Eppendorf, Martinistrasse 52, 20246 Hamburg, Germany

Contents:

Figure S1: Partial decomposition of compound **2** S2

Figure S2. Comparison of phosphonoacetate analogue **1** with ADPR S2

^{1}H-NMR, ^{13}C-NMR and ^{31}P-NMR data for compounds **1**, **2** and **5-10** S3

HPLC and MS data for final compound **1** S10

HPLC and MS data for final compound **2** S17

Figure S1: Partial decomposition of compound **2** in TEAB (0.1M), after 16 hours

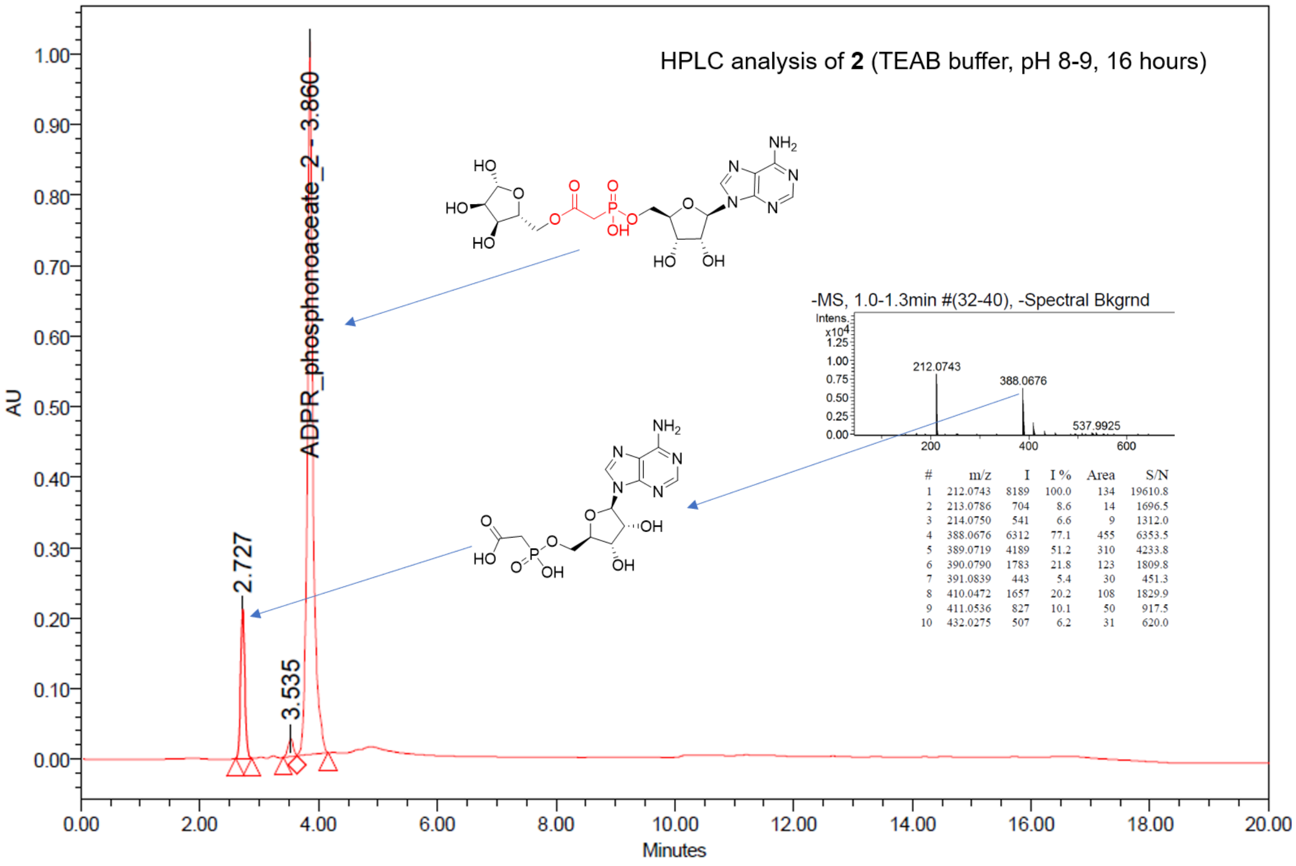

#	m/z	I	I %	Area	S/N
1	212.0743	8189	100.0	134	19610.8
2	213.0786	704	8.6	14	1696.5
3	214.0750	541	6.6	9	1312.0
4	388.0676	6312	77.1	455	6353.5
5	389.0719	4189	51.2	310	4233.8
6	390.0790	1783	21.8	123	1809.8
7	391.0839	443	5.4	30	451.3
8	410.0472	1657	20.2	108	1829.9
9	411.0536	827	10.1	50	917.5
10	432.0275	507	6.2	31	620.0

2

Figure S2. Comparison of phosphonoacetate analogue **1** with ADPR

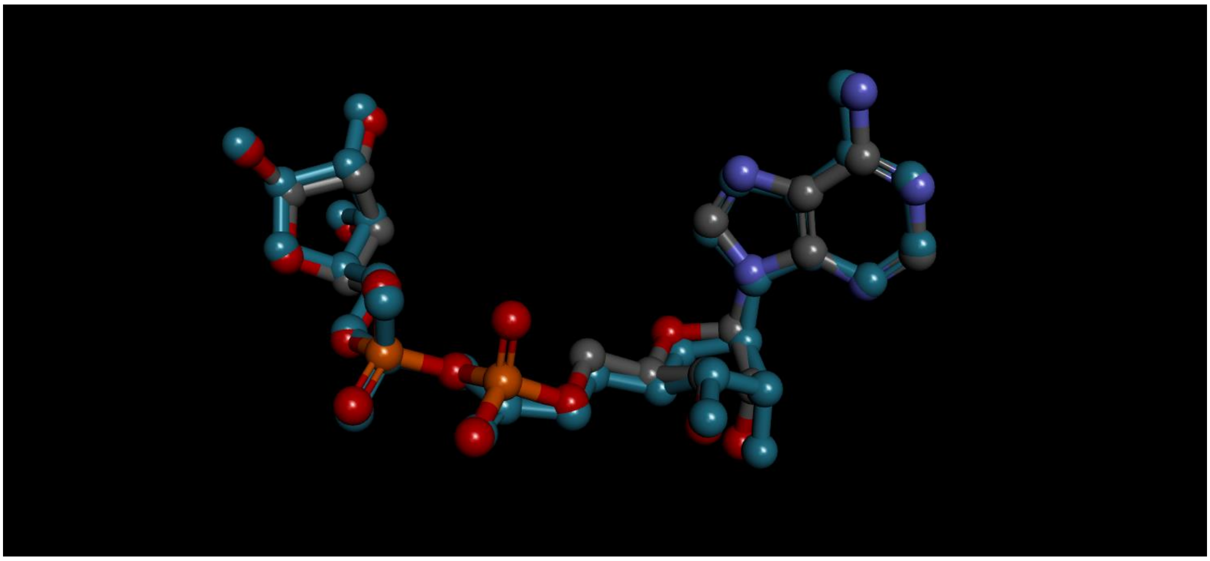

Superposition of **1** (all atoms shown in blue) with ADPR (coloured), Discovery studio (Accelrys)

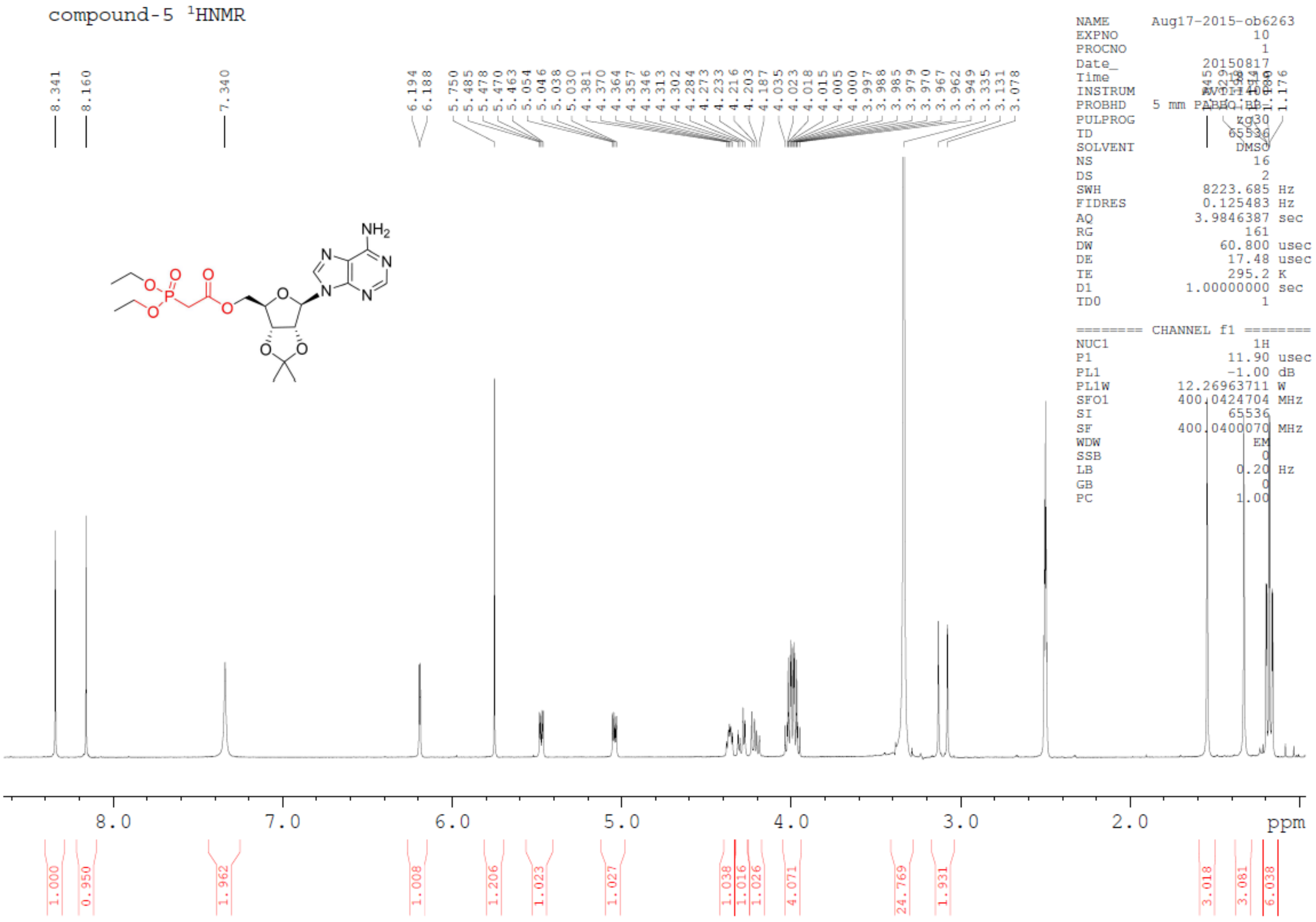
compound-5 1HNMR
8.341
8.160
7.340
6.194
6.188
5.750
5.485
5.478
5.470
5.463
5.054
5.046
5.038
5.030
4.381
4.370
4.364
4.357
4.346
4.313
4.302
4.284
4.273
4.233
4.216
4.203
4.187
4.035
4.023
4.018
4.015
4.005
4.000
3.997
3.988
3.985
3.979
3.970
3.967
3.962
3.949
3.335
3.131
3.078
1.176
NH2
N
O
P
NAME Aug17-2015-ob6263
EXPNO 10
PROCNO 1
Date_ 20150817
INSTRUM AVIII400
PROBHD 5 mm PABBO BB-
PULPROG zg30
TD 65536
SOLVENT DMSO
NS 16
DS 2
SWH 8223.685 Hz
FIDRES 0.125483 Hz
AQ 3.9846387 sec
RG 161
DW 60.800 usec
DE 17.48 usec
TE 295.2 K
D1 1.00000000 sec
TD0 1
======= CHANNEL f1 =======
NUC1 1H
P1 11.90 usec
PL1 -1.00 dB
PL1W 12.26963711 W
SFO1 400.0424704 MHz
SI 65536
SF 400.0400070 MHz
WDW EM
SSB 0
LB 0.20 Hz
GB 0
PC 1.00
8.0
7.0
6.0
5.0
4.0
3.0
2.0
ppm
1.000
0.950
1.962
1.008
1.206
1.023
1.027
1.038
1.016
1.026
4.071
24.769
1.931
3.018
3.081
6.038

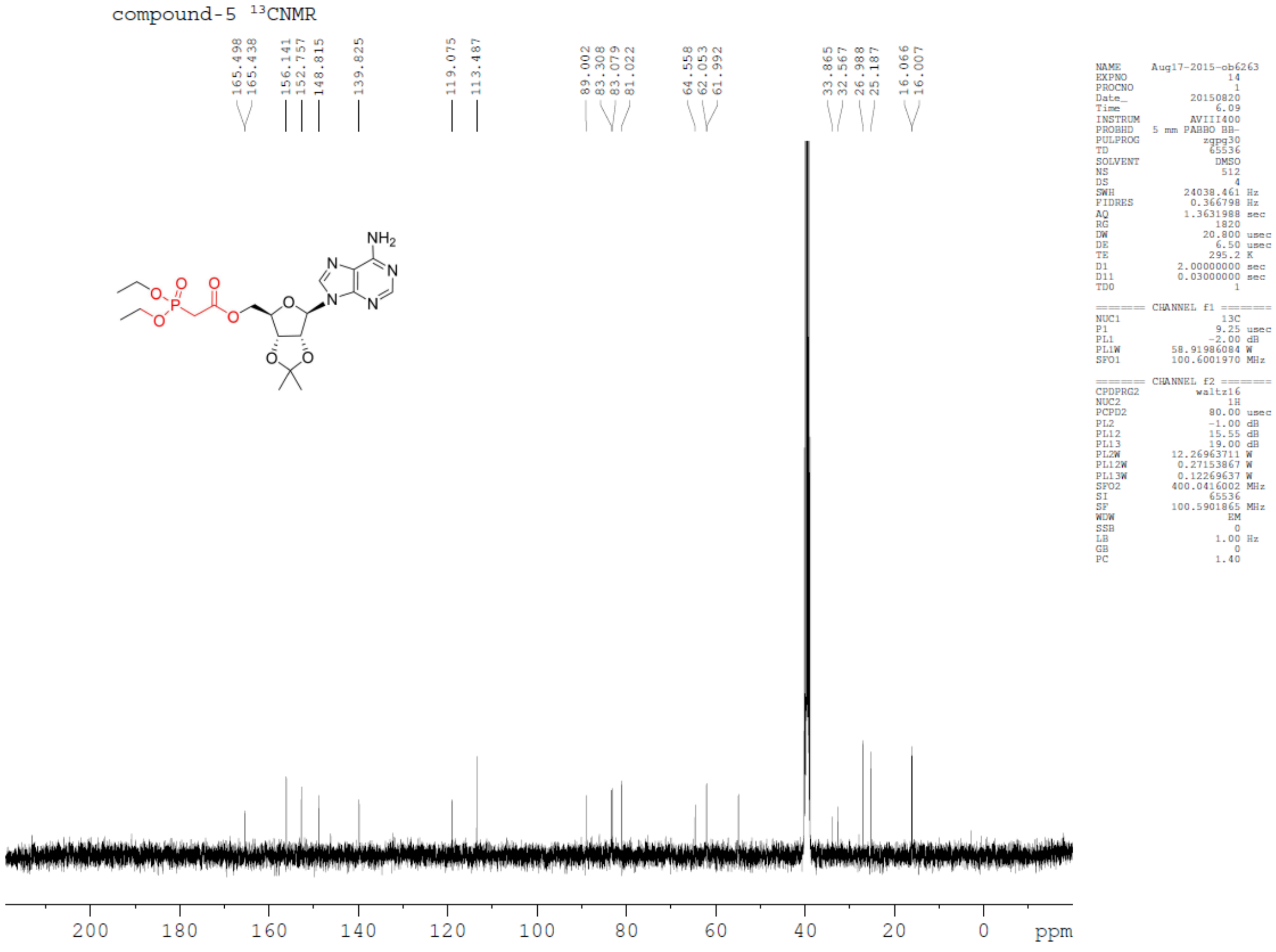
compound-5 13CNMR
165.498
165.438
156.141
152.757
148.815
139.825
119.075
113.487
89.002
83.308
83.079
81.022
64.558
62.053
61.992
33.865
32.567
26.988
25.187
16.066
16.007
NH2
N
O
P
NAME Aug17-2015-ob6263
EXPNO 14
PROCNO 1
Date_ 20150820
Time 6.09
INSTRUM AVIII400
PROBHD 5 mm PABBO BB-
PULPROG zgpg30
TD 65536
SOLVENT DMSO
NS 512
DS 4
SWH 24038.461 Hz
FIDRES 0.366798 Hz
AQ 1.3631988 sec
RG 1820
DW 20.800 usec
DE 6.50 usec
TE 295.2 K
D1 2.00000000 sec
D11 0.03000000 sec
TD0 1
====== CHANNEL f1 ======
NUC1 13C
P1 9.25 usec
PL1 -2.00 dB
PL1W 58.91986084 W
SFO1 100.6001970 MHz
====== CHANNEL f2 ======
CPDPRG2 waltz16
NUC2 1H
PCPD2 80.00 usec
PL2 -1.00 dB
PL12 15.55 dB
PL13 19.00 dB
PL2W 12.26963711 W
PL12W 0.27153867 W
PL13W 0.12269637 W
SFO2 400.0416002 MHz
SI 65536
SF 100.5901865 MHz
WDW EM
SSB 0
LB 1.00 Hz
GB 0
PC 1.40
200
180
160
140
120
100
80
60
40
20
0
ppm

compound-5 ^{31}PNMR

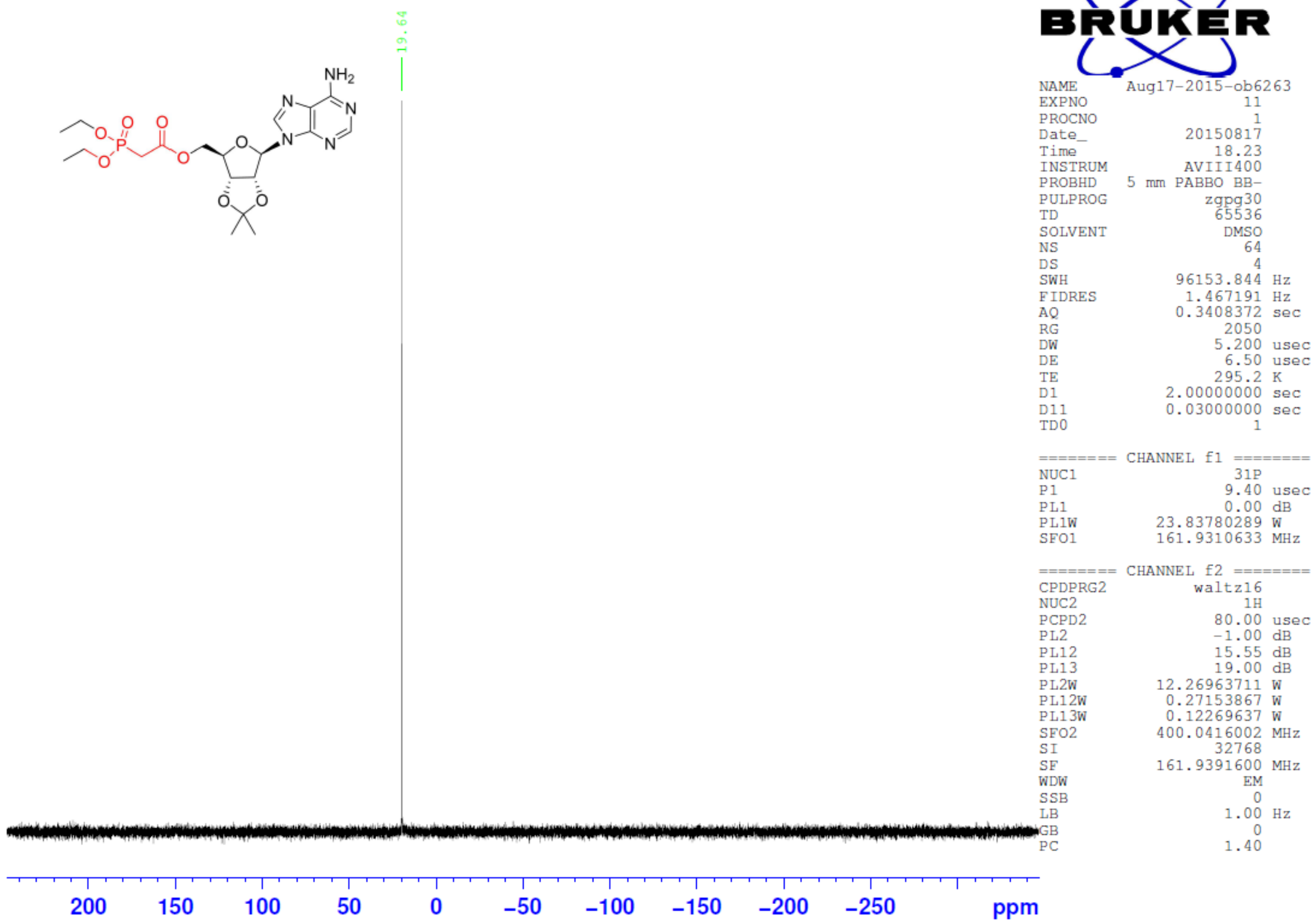

compound-6 ^{1}HNMR

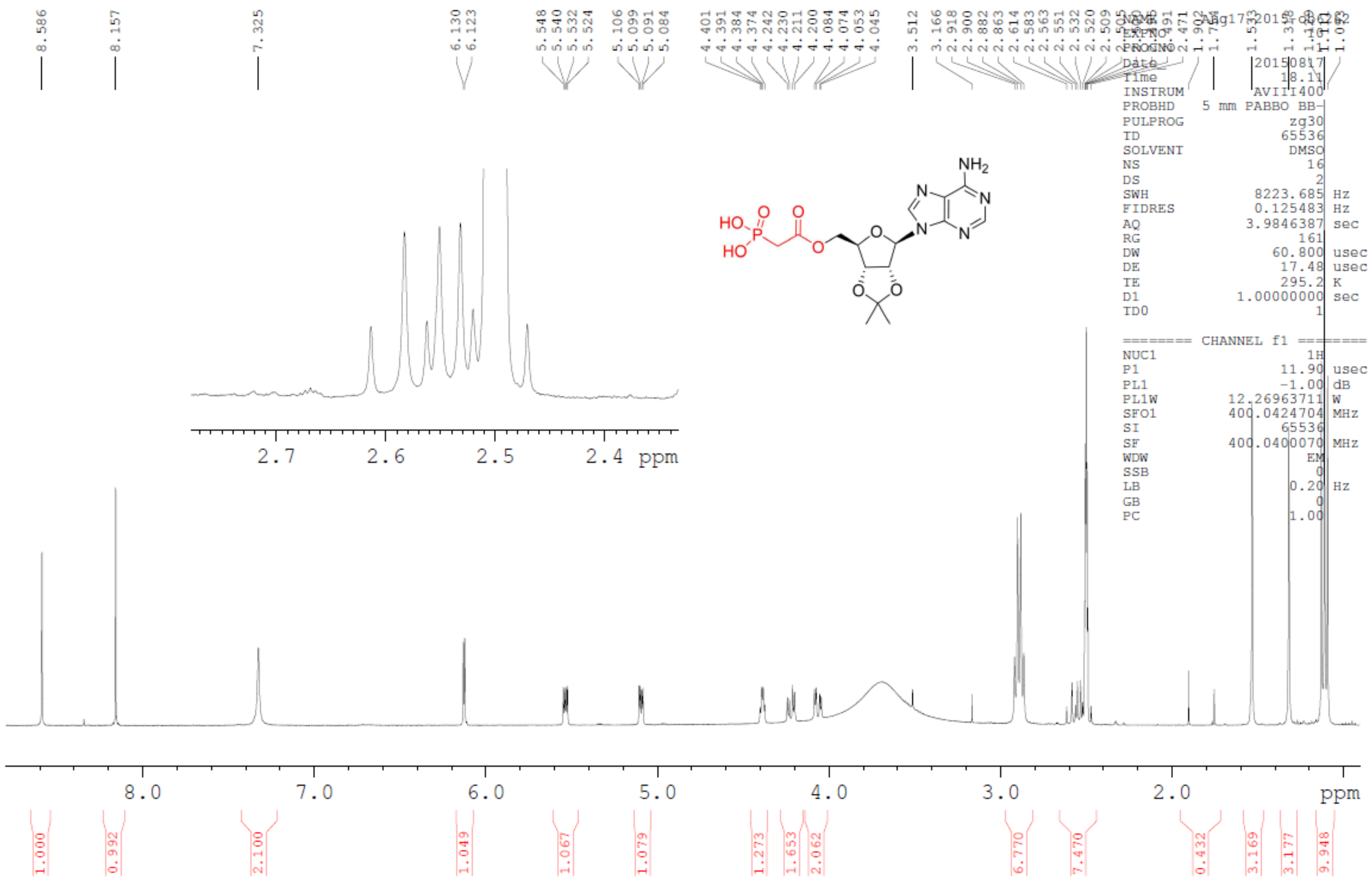

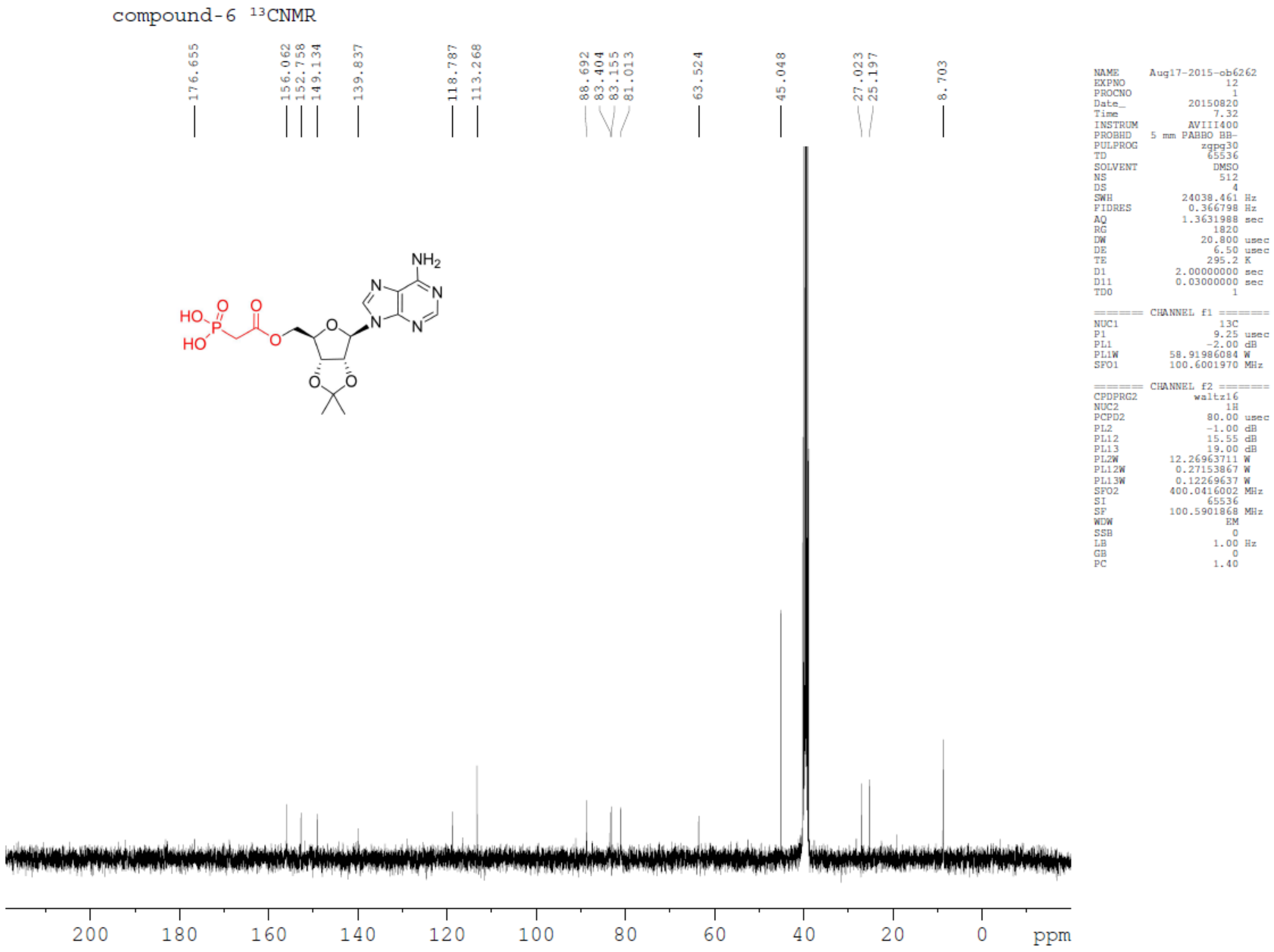
compound-6 13CNMR
176.655
156.062
152.758
149.134
139.837
118.787
113.268
88.692
83.404
83.155
81.013
63.524
45.048
27.023
25.197
8.703
NH2
N
N
N
N
O
HO
P
HO
O
O
O
O
O
O
NAME Aug17-2015-ob6262
EXPNO 12
PROCNO 1
Date_ 20150820
Time 7.32
INSTRUM AVIII400
PROBHD 5 mm PABBO BB-
PULPROG zgpg30
TD 65536
SOLVENT DMSO
NS 512
DS 4
SWH 24038.461 Hz
FIDRES 0.366798 Hz
AQ 1.3631988 sec
RG 1820
DW 20.800 usec
DE 6.50 usec
TE 295.2 K
D1 2.00000000 sec
D11 0.03000000 sec
TD0 1
====== CHANNEL f1 ======
NUC1 13C
P1 9.25 usec
PL1 -2.00 dB
PL1W 58.91986084 W
SFO1 100.6001970 MHz
====== CHANNEL f2 ======
CPDPRG2 waltz16
NUC2 1H
PCPD2 80.00 usec
PL2 -1.00 dB
PL12 15.55 dB
PL13 19.00 dB
PL2W 12.26963711 W
PL12W 0.27153867 W
PL13W 0.12269637 W
SFO2 400.0416002 MHz
SI 65536
SF 100.5901868 MHz
WDW EM
SSB 0
LB 1.00 Hz
GB 0
PC 1.40
200
180
160
140
120
100
80
60
40
20
0
ppm

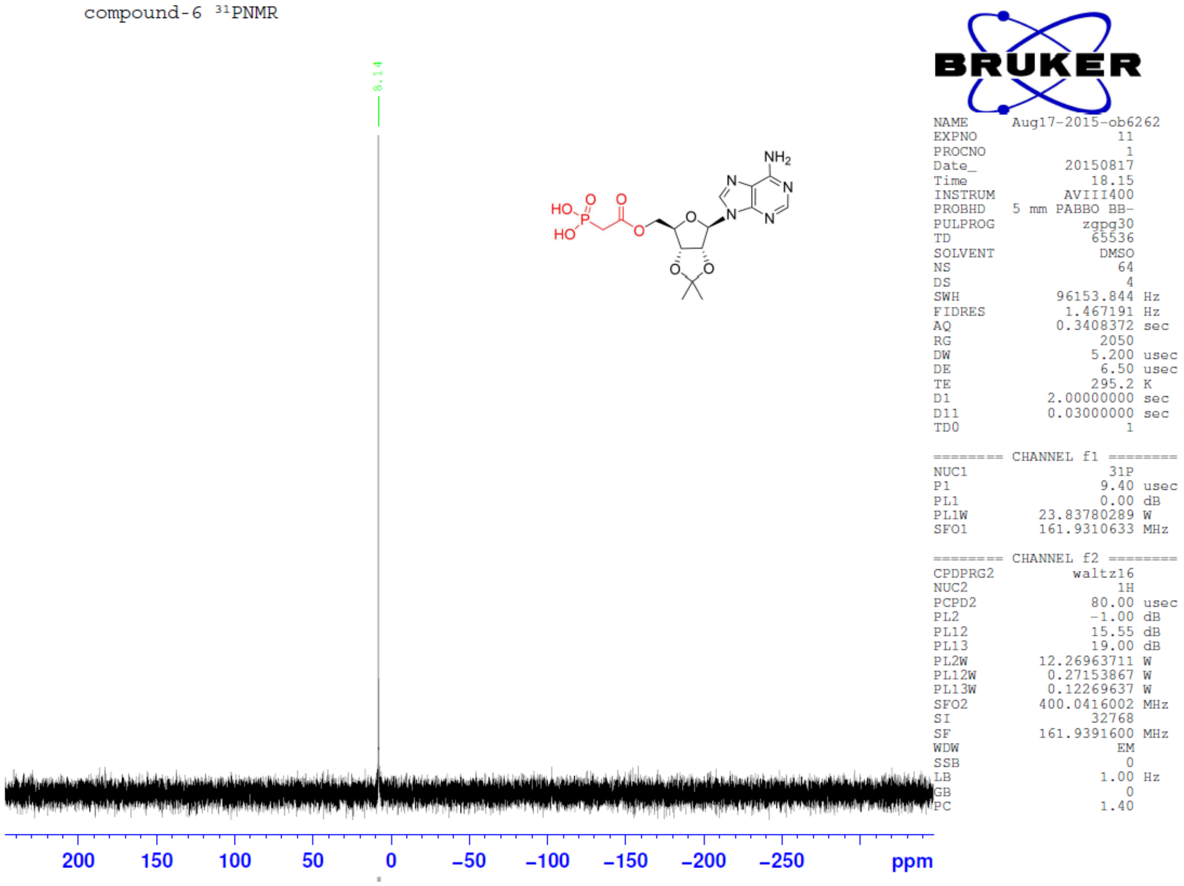
compound-6 31PNMR
8.14
NH2
N
N
N
N
O
HO
P
HO
O
O
O
O
O
O
BRUKER
NAME Aug17-2015-ob6262
EXPNO 11
PROCNO 1
Date_ 20150817
Time 18.15
INSTRUM AVIII400
PROBHD 5 mm PABBO BB-
PULPROG zgpg30
TD 65536
SOLVENT DMSO
NS 64
DS 4
SWH 96153.844 Hz
FIDRES 1.467191 Hz
AQ 0.3408372 sec
RG 2050
DW 5.200 usec
DE 6.50 usec
TE 295.2 K
D1 2.00000000 sec
D11 0.03000000 sec
TD0 1
======== CHANNEL f1 ========
NUC1 31P
P1 9.40 usec
PL1 0.00 dB
PL1W 23.83780289 W
SFO1 161.9310633 MHz
======== CHANNEL f2 ========
CPDPRG2 waltz16
NUC2 1H
PCPD2 80.00 usec
PL2 -1.00 dB
PL12 15.55 dB
PL13 19.00 dB
PL2W 12.26963711 W
PL12W 0.27153867 W
PL13W 0.12269637 W
SFO2 400.0416002 MHz
SI 32768
SF 161.9391600 MHz
WDW EM
SSB 0
LB 1.00 Hz
GB 0
PC 1.40
200
150
100
50
0
-50
-100
-150
-200
-250
ppm

compound-7 ^{1}HNMR

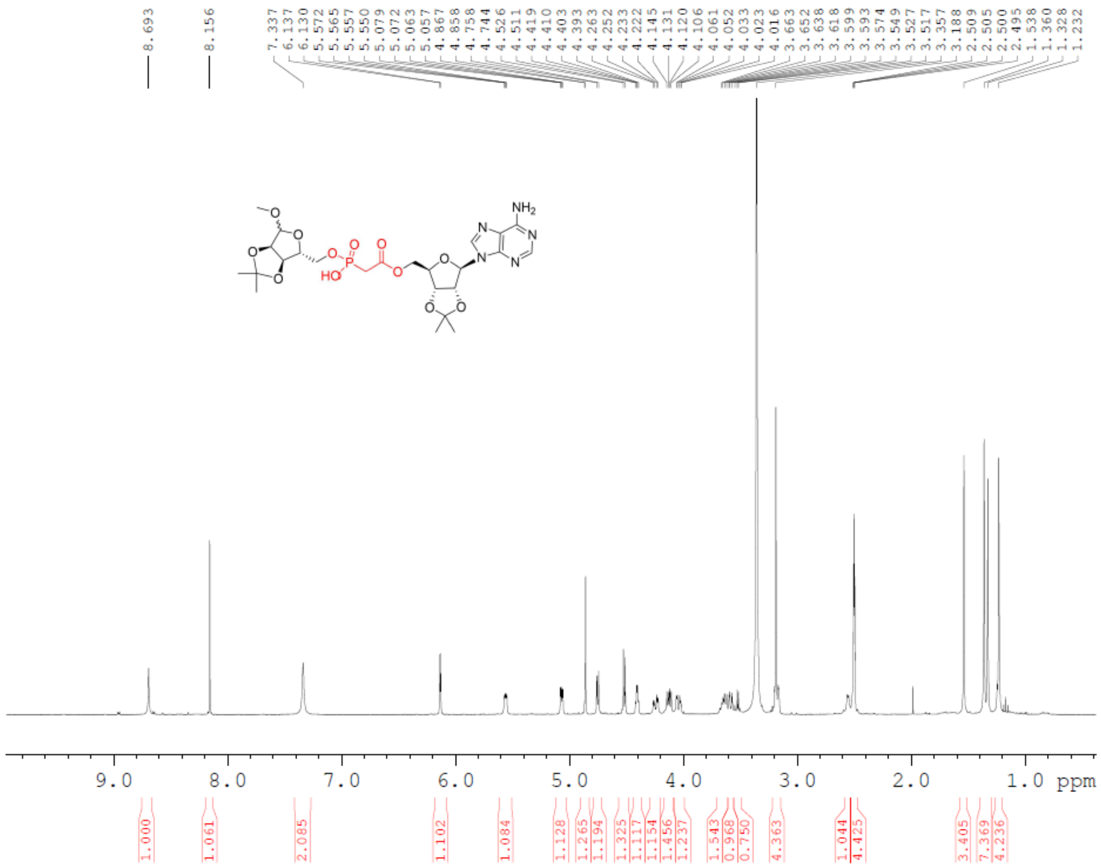

NAME	Aug17-2015-ob6261
EXPNO	10
PROCNO	1
Date_	20150817
Time	18.03
INSTRUM	AVIII400
PROBHD	5 mm PABBO BB-
PULPROG	zg30
TD	65536
SOLVENT	DMSO
NS	16
DS	2
SWH	8223.685 Hz
FIDRES	0.125483 Hz
AQ	3.9846387 sec
RG	144
DW	60.800 usec
DE	17.48 usec
TE	295.2 K
D1	1.00000000 sec
TD0	1
======== CHANNEL f1 ========	
NUC1	1H
P1	11.90 usec
PL1	-1.00 dB
PL1W	12.26963711 W
SFO1	400.0424704 MHz
SI	65536
SF	400.0400070 MHz
WDW	EM
SSB	0
LB	0.20 Hz
GB	0
PC	1.00

compound-7 ^{13}CNMR

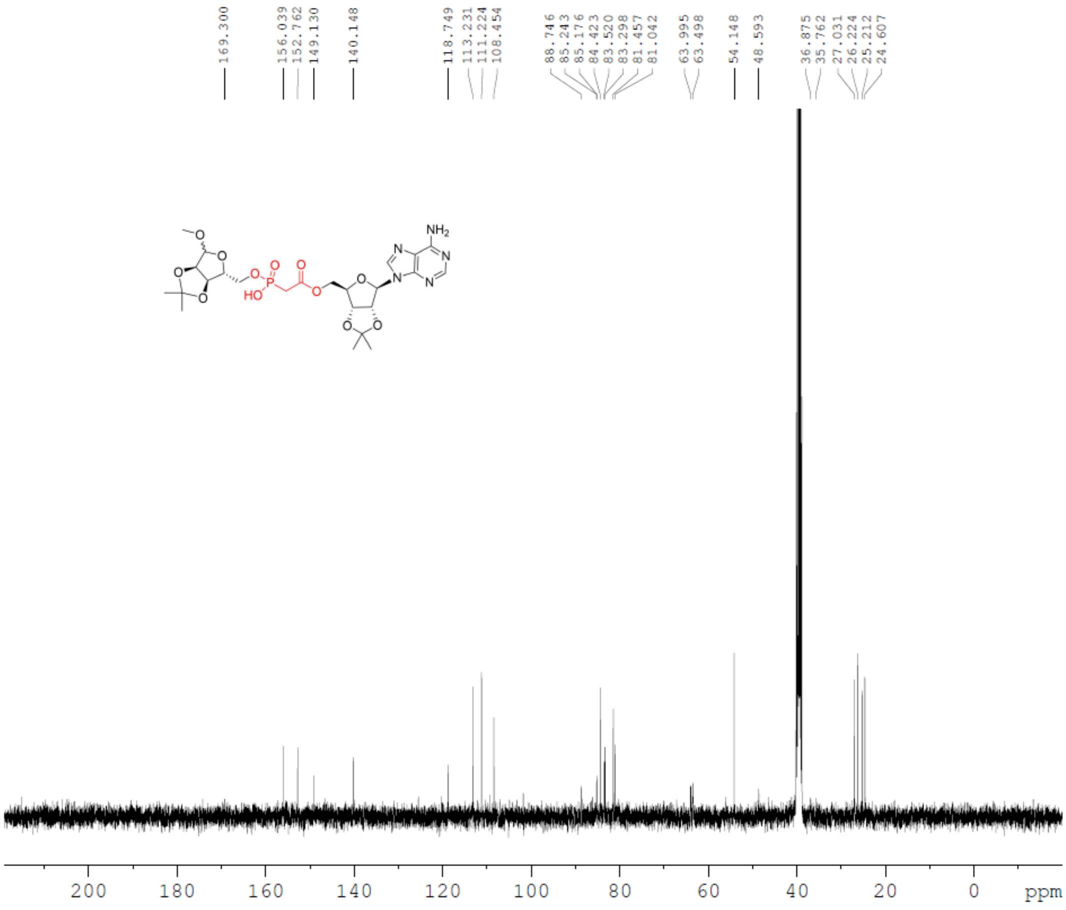

NAME	Aug17-2015-ob6261
EXPNO	13
PROCNO	1
Date_	20150817
Time	21.31
INSTRUM	AVIII400
PROBHD	5 mm PABBO BB-
PULPROG	zgpg30
TD	65536
SOLVENT	DMSO
NS	512
DS	4
SWH	24038.461 Hz
FIDRES	0.366798 Hz
AQ	1.3631988 sec
RG	1820
DW	20.800 usec
DE	6.50 usec
TE	295.2 K
D1	2.00000000 sec
D11	0.03000000 sec
TD0	1
====== CHANNEL f1 ======	
NUC1	13C
P1	9.25 usec
PL1	-2.00 dB
PL1W	58.91986084 W
SFO1	100.6001970 MHz
====== CHANNEL f2 ======	
CPDPRG2	waltz16
NUC2	1H
PCPD2	80.00 usec
PL2	-1.00 dB
PL12	15.55 dB
PL13	19.00 dB
PL2W	12.26963711 W
PL12W	0.27153867 W
PL13W	0.12269637 W
SFO2	400.0416002 MHz
SI	65536
SF	100.5901857 MHz
WDW	EM
SSB	0
LB	1.00 Hz
GB	0
PC	1.40

compound-7 ^{31}PNMR

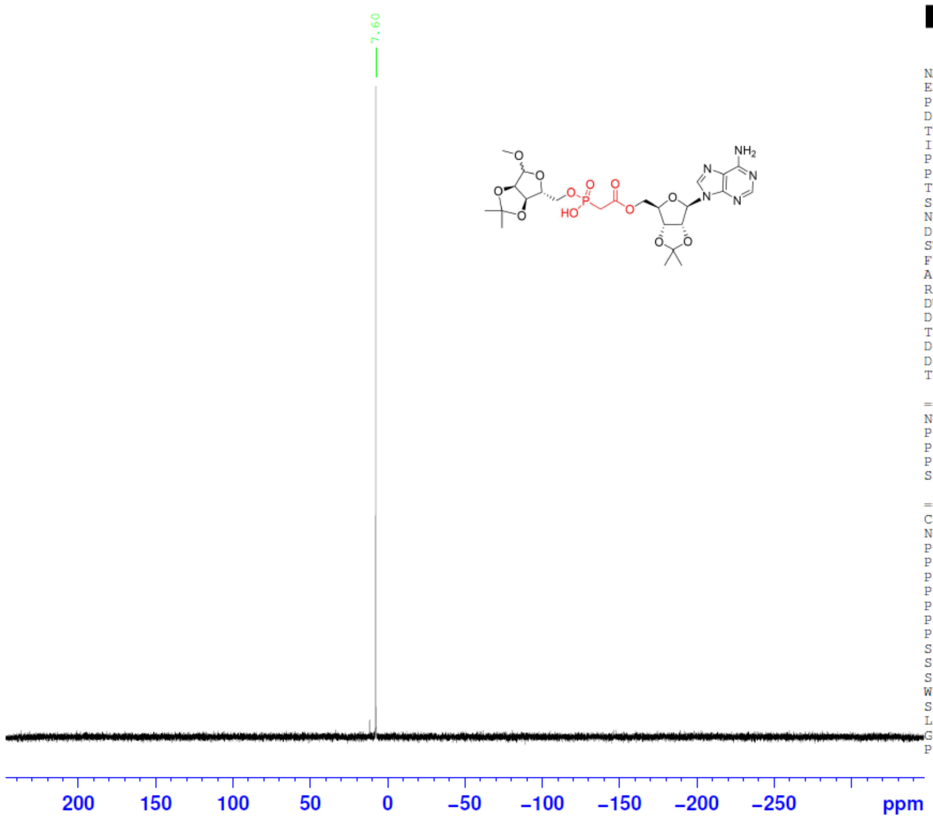


```
NAME       Aug17-2015-ob6261
EXPNO                     11
PROCNO                     1
Date_               20150817
Time                   18.07
INSTRUM             AVIII400
PROBHD     5 mm PABBO BB-
PULPROG               zgpg30
TD                     65536
SOLVENT                 DMSO
NS                        64
DS                         4
SWH               96153.844 Hz
FIDRES             1.467191 Hz
AQ                0.3408372 sec
RG                      2050
DW                     5.200 usec
DE                      6.50 usec
TE                     295.2 K
D1                2.00000000 sec
D11               0.03000000 sec
TD0                        1

======== CHANNEL f1 ========
NUC1                     31P
P1                      9.40 usec
PL1                     0.00 dB
PL1W             23.83780289 W
SFO1             161.9310633 MHz

======== CHANNEL f2 ========
CPDPRG2              waltz16
NUC2                      1H
PCPD2                  80.00 usec
PL2                    -1.00 dB
PL12                   15.55 dB
PL13                   19.00 dB
PL2W             12.26963711 W
PL12W             0.27153867 W
PL13W             0.12269637 W
SFO2             400.0416002 MHz
SI                     32768
SF               161.9391600 MHz
WDW                       EM
SSB                        0
LB                      1.00 Hz
GB                         0
PC                      1.40
```

compound-1 ^{1}HNMR (DOSY)

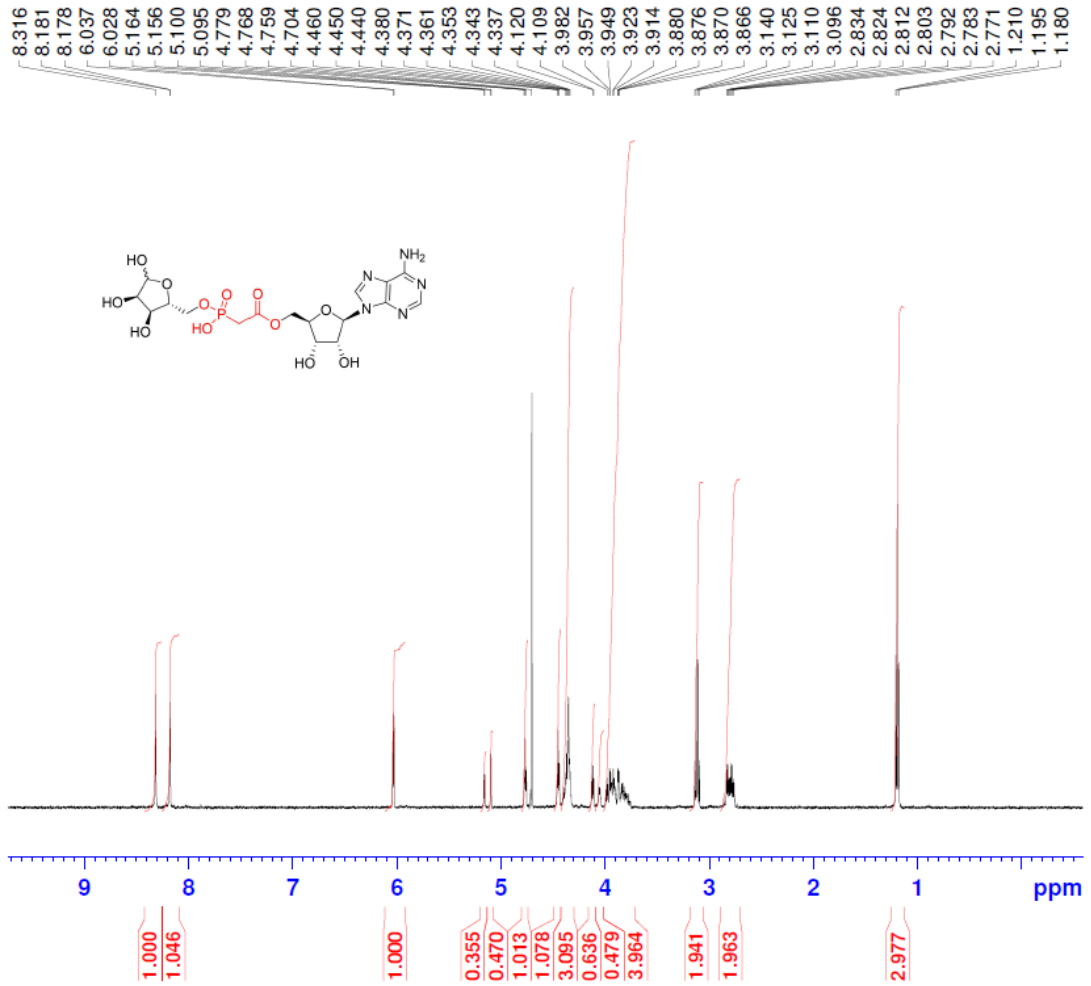


```
NAME                   OBB44
EXPNO                     15
PROCNO                     1
Date_               20151119
Time                   17.29
INSTRUM                spect
PROBHD     5 mm PABBO BB-
PULPROG          ledbpgp2s1d
TD                     65536
SOLVENT                  D2O
NS                        16
DS                         2
SWH               10330.578 Hz
FIDRES             0.157632 Hz
AQ                3.1719923 sec
RG                       406
DW                    48.400 usec
DE                      9.73 usec
TE                     298.2 K
D1                3.00000000 sec
D16               0.00020000 sec
D20               0.05000000 sec
D21               0.00500000 sec
TD0                        1

======== CHANNEL f1 ========
NUC1                      1H
P1                     10.00 usec
P2                     20.00 usec
PL1                    -0.12 dB
PL1W             19.35150909 W
SFO1             500.1330885 MHz

====== GRADIENT CHANNEL =====
GPNAM6              sine.100
GPNAM7              sine.100
```

compound-1 ^{13}CNMR

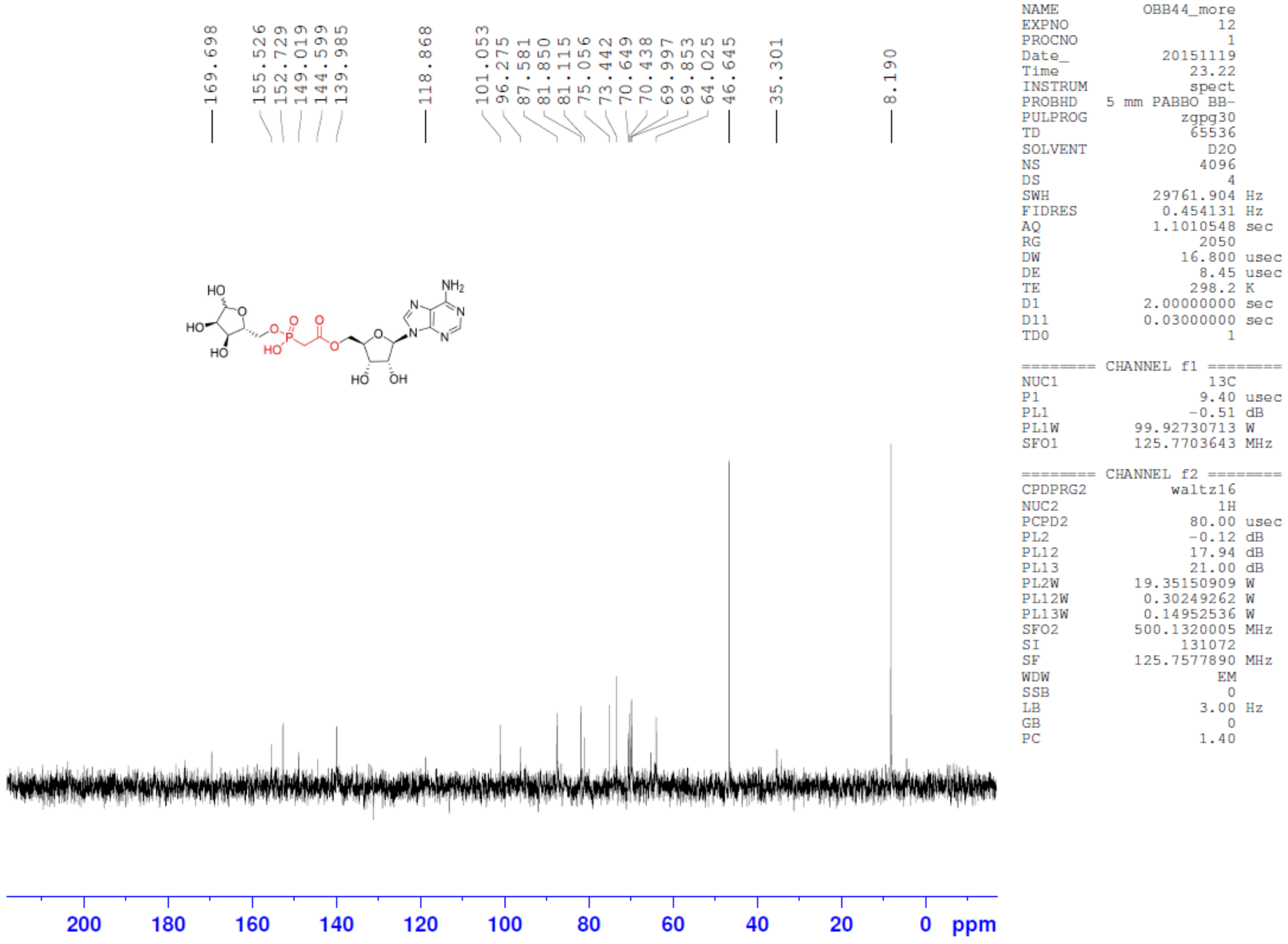

compound-1 ^{31}PNMR

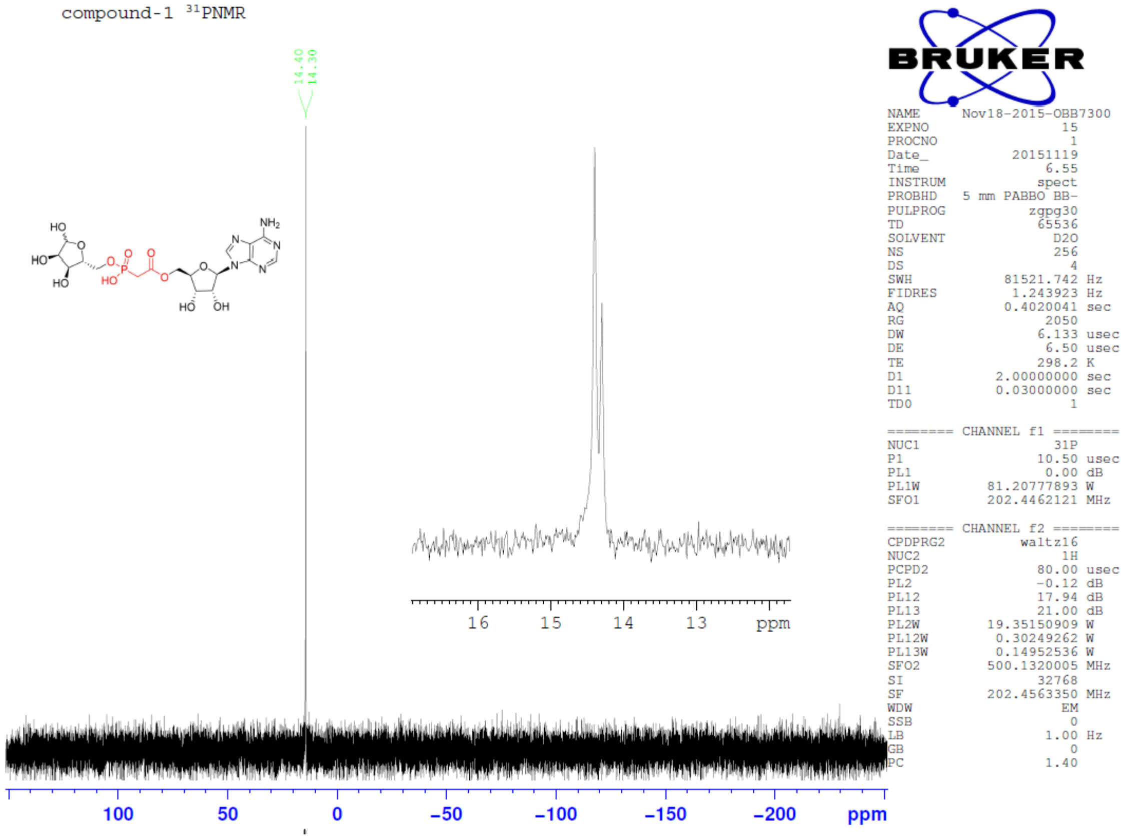

compound-1 HSQC

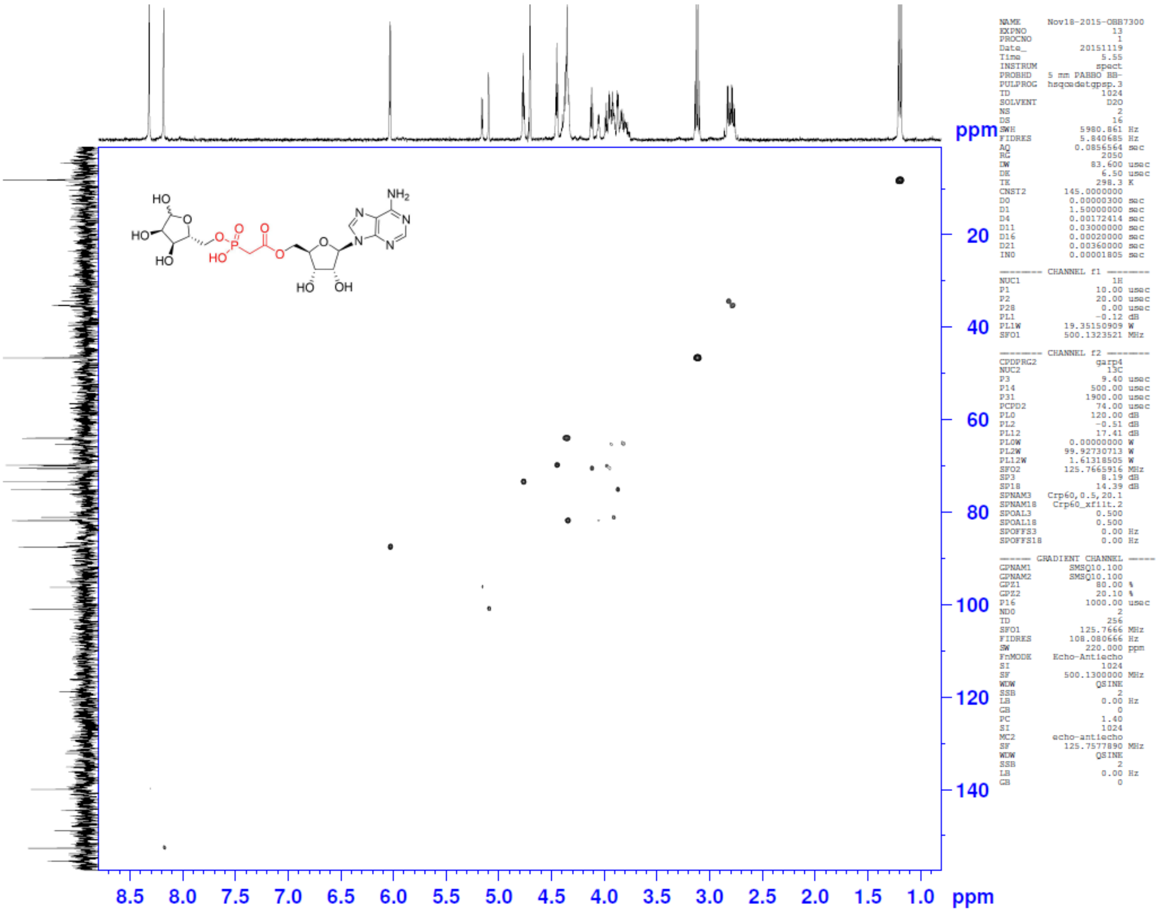

compound-1 HMBC

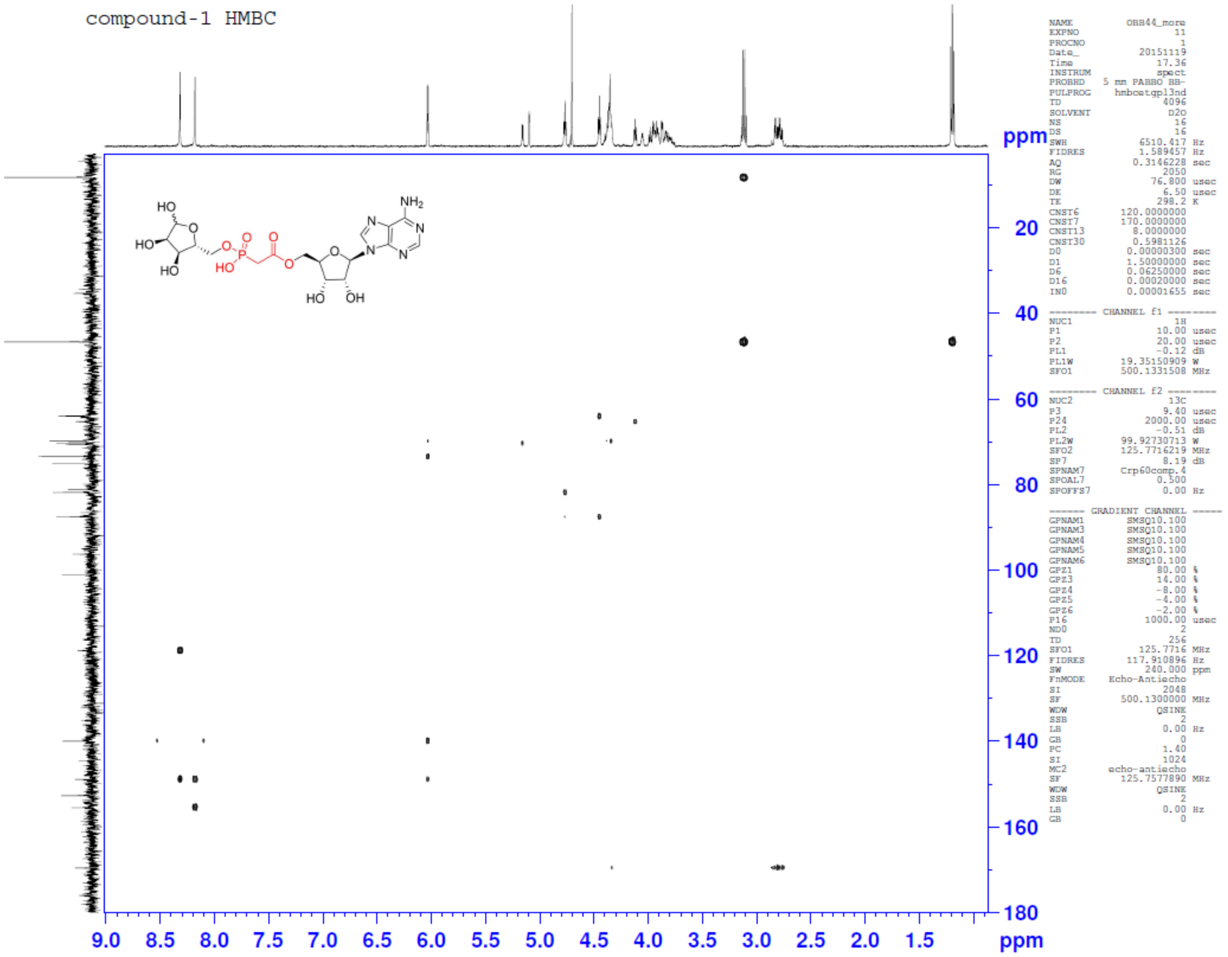

Spectrum Index Plot

Compound-1 UV/HPLC

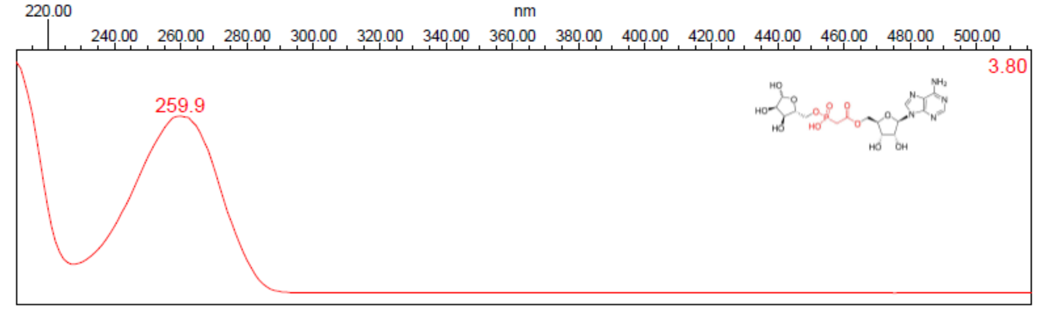

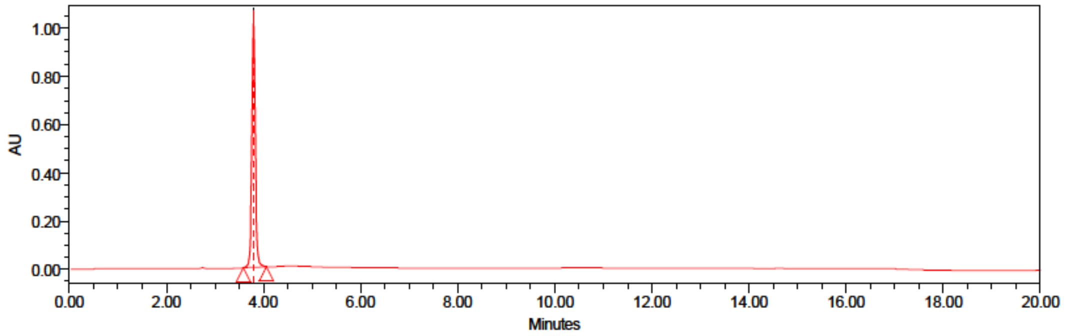

PDA Result Table

	Name	RT	Area	Purity (#1) Angle	Purity (#1) Threshold	Purity Flag	Match (#1) Spect. Name	Match (#1) Angle	Match (#1) Threshold	PDA Match Flag
1	Compound-1	3.798	5141072			No				No

Confirmation of Expected Formula

Sample-ID	compound-1	Submitter	Ondrej Baszczynski
Analysis Name	po_ob_obb44_345634_43_01_50210.d	Supervisor	Barry Potter
Method used	Confirm Formula Negative 50to1500 loop inj.m	Acquisition Date	26/11/2015 10:33:36
Ionisation Mode	negative electrospray (ESI)		

-MS, 1.0-1.3min #(32-40), -Spectral Bkgrnd

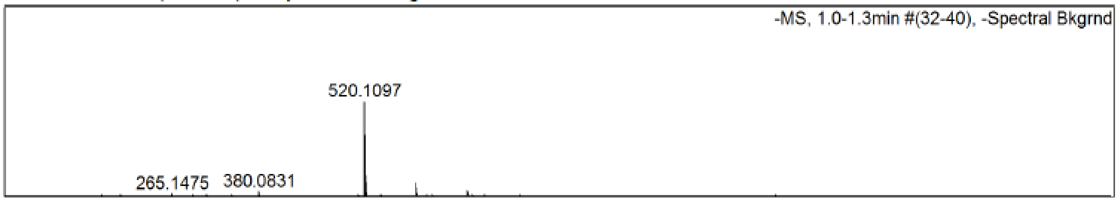

#	m/z	I	I %	Area	S/N
1	265.1475	142	2.2	6	3780.9
2	380.0831	328	5.0	20	14138.4
3	520.1097	6522	100.0	496	21988.3
4	520.9155	285	4.4	12	968.0
5	521.1132	1445	22.2	101	4923.3
6	522.1150	292	4.5	22	1006.4
7	588.0950	939	14.4	78	8695.3
8	589.1039	239	3.7	14	2221.1
9	656.0836	395	6.0	32	4196.9
10	724.0699	140	2.1	10	2161.1

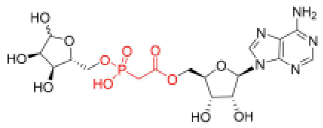

Generate Molecular Formula Parameters

Charge	Tolerance	SearchRadius	H/C Ratio min.	H/C Ratio max.	Electron Conf.	Nitrogen Rule	sigma limit
negative	10 ppm	0.05 m/z	0	3	both	true	0.05

Expected Formula C17 H24 N5 O12 P **Adduct(s):** H, Na

#	meas. m/z	theo. m/z	Err[ppm]	Sigma	Formula
1	520.1097	520.108083	-2.10	0.0078	C 17 H 23 N 5 O 12 P 1

compound-8 ^{1}HNMR

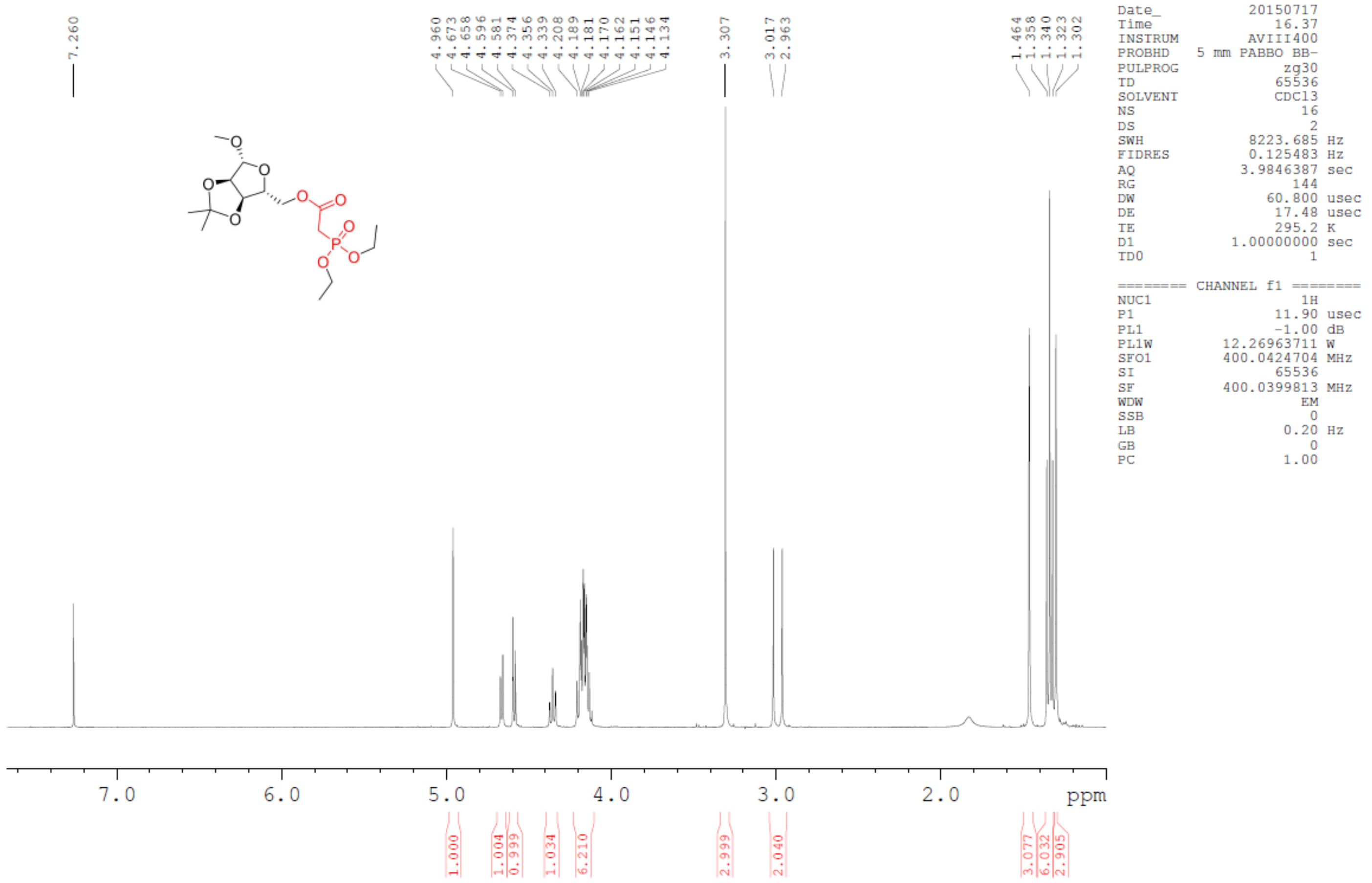

compound-8 ^{13}CNMR

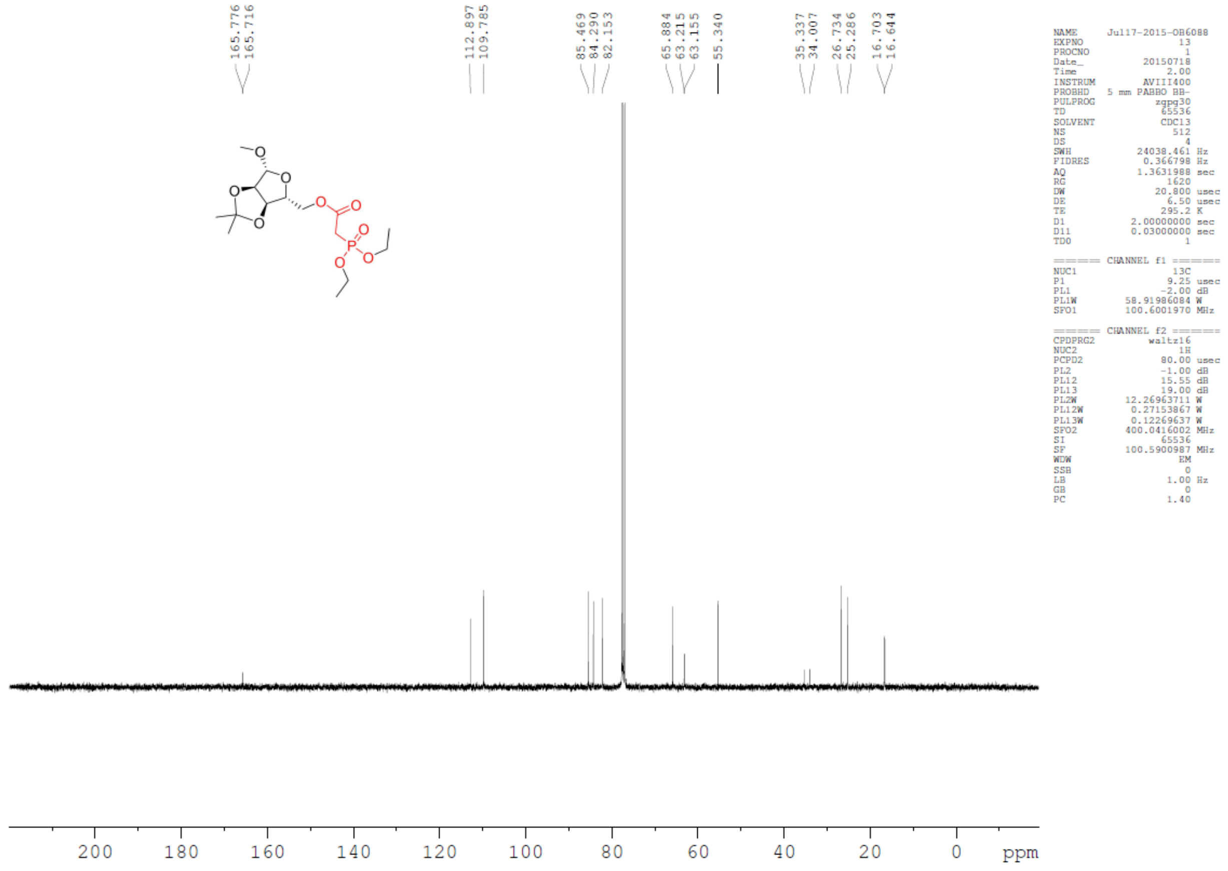

compound-8 ^{31}PNMR

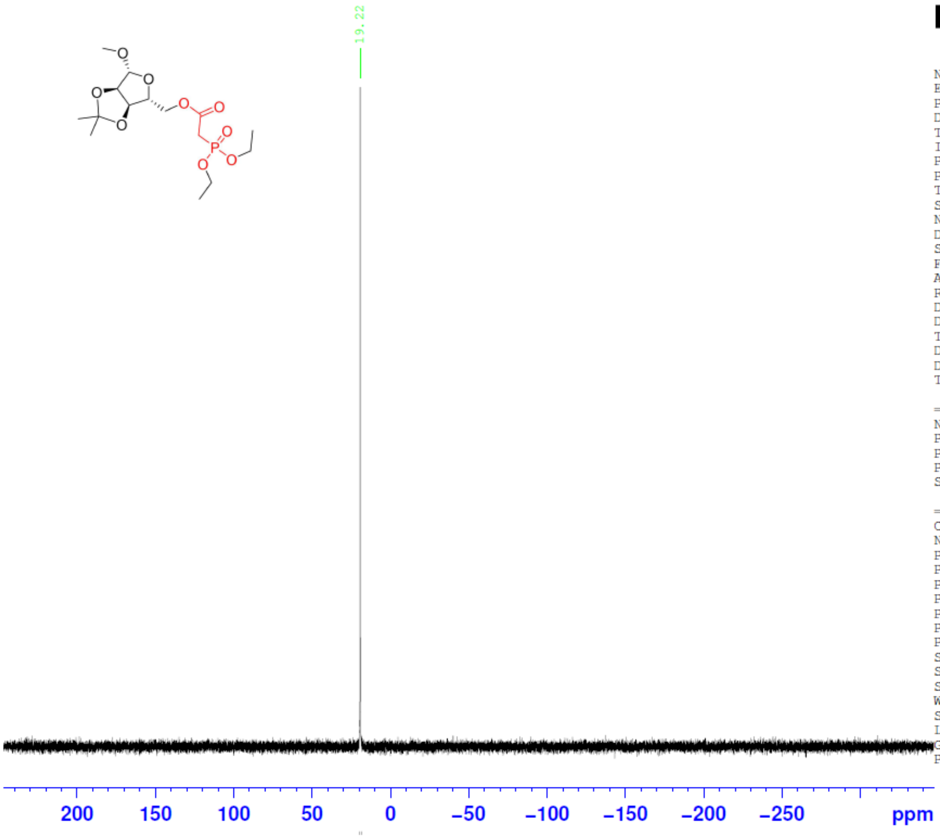

BRUKER

```
NAME        Jul17-2015-OB6088
EXPNO                      11
PROCNO                      1
Date_                20150717
Time                    16.40
INSTRUM              AVIII400
PROBHD      5 mm PABBO BB-
PULPROG                zgpg30
TD                      65536
SOLVENT                 CDCl3
NS                         64
DS                          4
SWH                 96153.844 Hz
FIDRES               1.467191 Hz
AQ                  0.3408372 sec
RG                       2050
DW                      5.200 usec
DE                       6.50 usec
TE                      295.2 K
D1                 2.00000000 sec
D11                0.03000000 sec
TD0                         1

======== CHANNEL f1 ========
NUC1                      31P
P1                       9.40 usec
PL1                      0.00 dB
PL1W              23.83780289 W
SFO1              161.9310633 MHz

======== CHANNEL f2 ========
CPDPRG2               waltz16
NUC2                       1H
PCPD2                   80.00 usec
PL2                     -1.00 dB
PL12                    15.55 dB
PL13                    19.00 dB
PL2W              12.26963711 W
PL12W              0.27153867 W
PL13W              0.12269637 W
SFO2              400.0416002 MHz
SI                      32768
SF                161.9391600 MHz
WDW                        EM
SSB                         0
LB                       1.00 Hz
GB                          0
PC                       1.40
```

compound-9 ^{1}HNMR

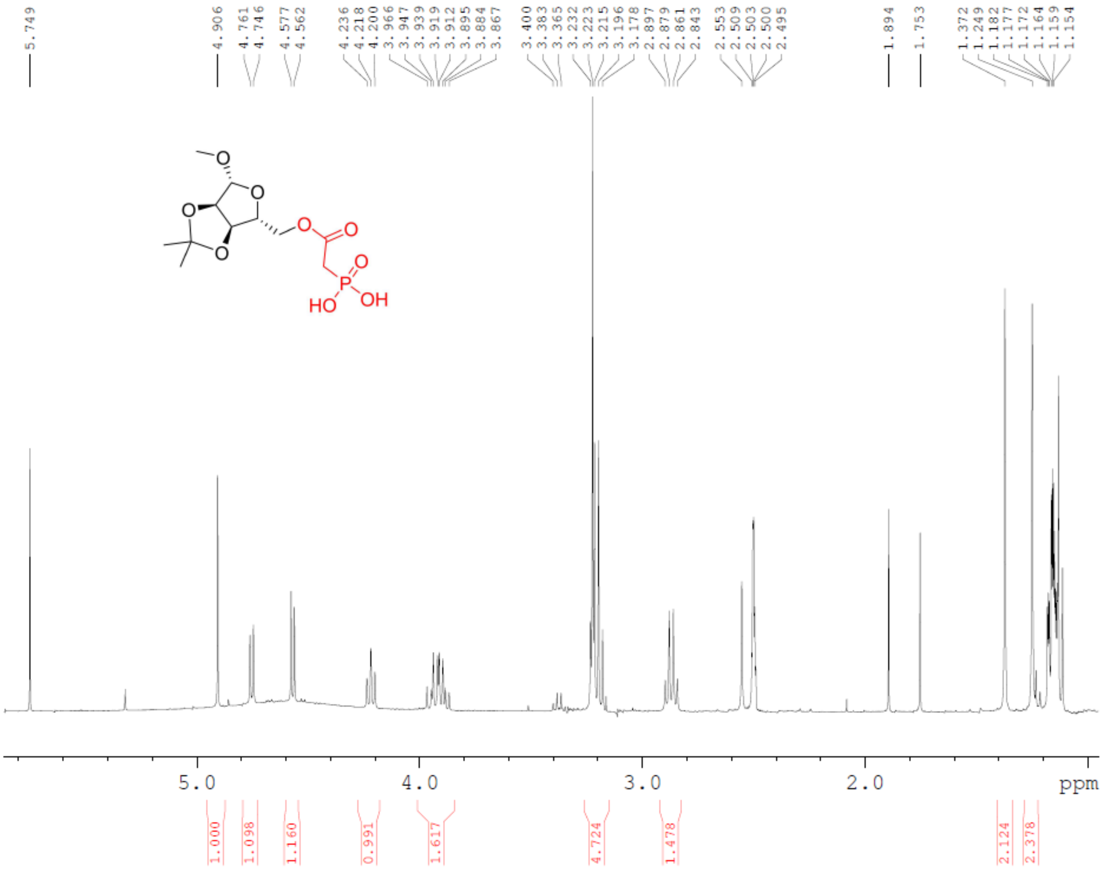


```
NAME        Aug10-2015-OB6081
EXPNO                      10
PROCNO                      1
Date_                20150810
Time                    11.22
INSTRUM              AVIII400
PROBHD      5 mm PABBO BB-
PULPROG                  zg30
TD                      65536
SOLVENT                  DMSO
NS                         16
DS                          2
SWH                  8223.685 Hz
FIDRES               0.125483 Hz
AQ                  3.9846387 sec
RG                        114
DW                     60.800 usec
DE                      17.48 usec
TE                      295.2 K
D1                 1.00000000 sec
TD0                         1

======== CHANNEL f1 ========
NUC1                       1H
P1                      11.90 usec
PL1                     -1.00 dB
PL1W              12.26963711 W
SFO1              400.0424704 MHz
SI                      65536
SF                400.0400070 MHz
WDW                        EM
SSB                         0
LB                       0.20 Hz
GB                          0
PC                       1.00
```

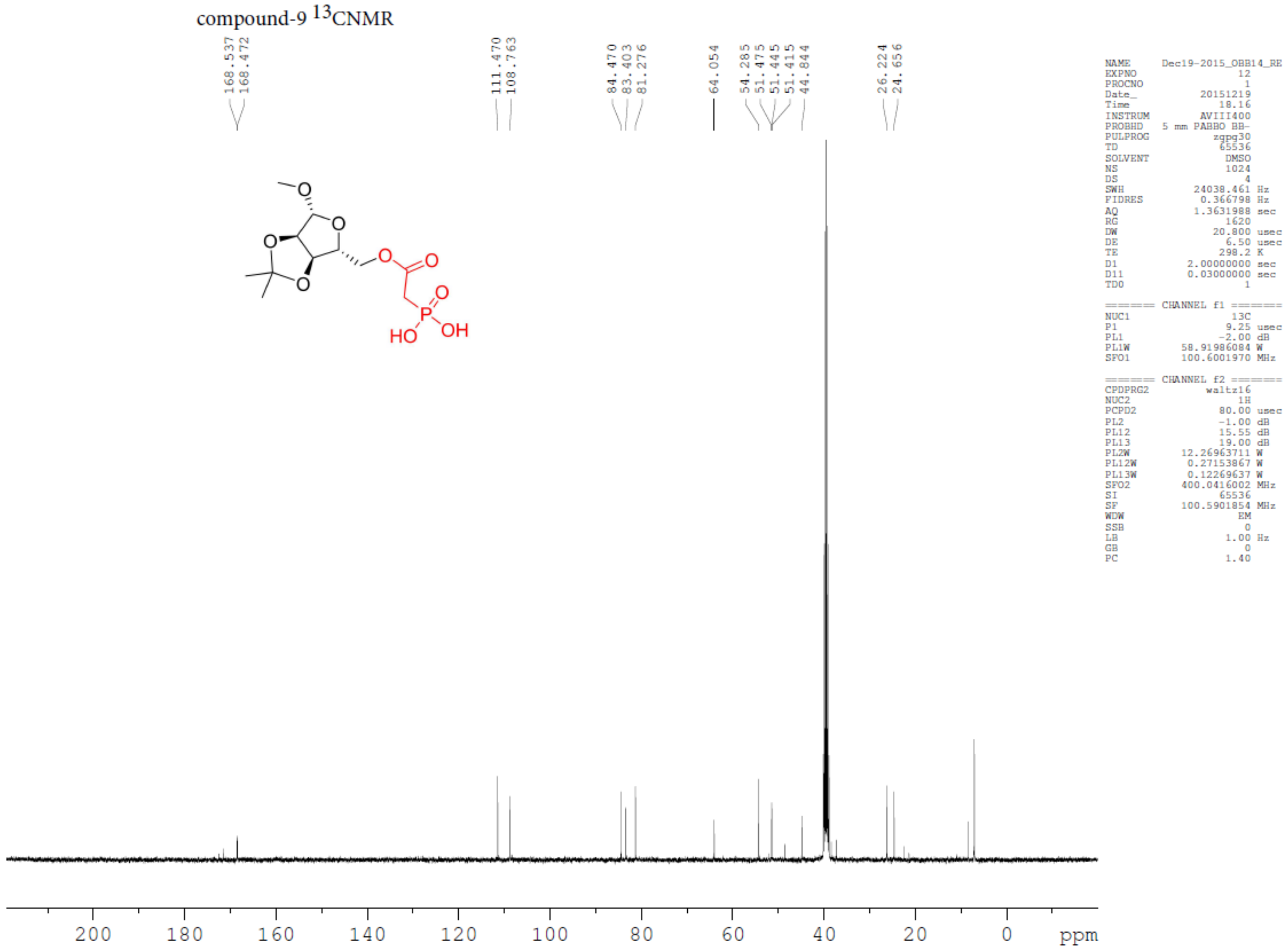
compound-9 13CNMR
168.537
168.472
111.470
108.763
84.470
83.403
81.276
64.054
54.285
51.475
51.445
51.415
44.844
26.224
24.656
O
O
O
O
O
O
O
P
HO
OH
NAME Dec19-2015_OBB14_RE
EXPNO 12
PROCNO 1
Date_ 20151219
Time 18.16
INSTRUM AVIII400
PROBHD 5 mm PABBO BB-
PULPROG zgpg30
TD 65536
SOLVENT DMSO
NS 1024
DS 4
SWH 24038.461 Hz
FIDRES 0.366798 Hz
AQ 1.3631988 sec
RG 1620
DW 20.800 usec
DE 6.50 usec
TE 298.2 K
D1 2.00000000 sec
D11 0.03000000 sec
TD0 1
====== CHANNEL f1 ======
NUC1 13C
P1 9.25 usec
PL1 -2.00 dB
PL1W 58.91986084 W
SFO1 100.6001970 MHz
====== CHANNEL f2 ======
CPDPRG2 waltz16
NUC2 1H
PCPD2 80.00 usec
PL2 -1.00 dB
PL12 15.55 dB
PL13 19.00 dB
PL2W 12.26963711 W
PL12W 0.27153867 W
PL13W 0.12269637 W
SFO2 400.0416002 MHz
SI 65536
SF 100.5901854 MHz
WDW EM
SSB 0
LB 1.00 Hz
GB 0
PC 1.40
200
180
160
140
120
100
80
60
40
20
0
ppm

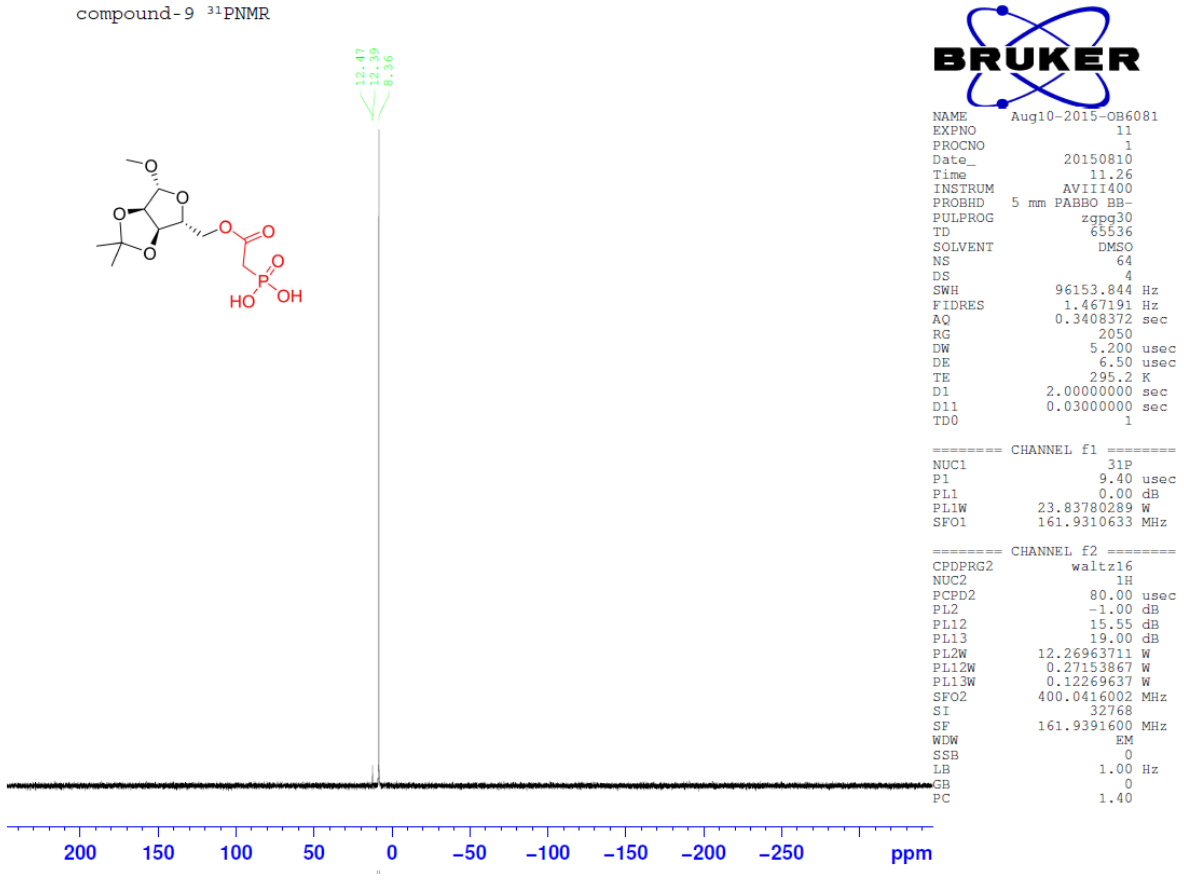
compound-9 31PNMR
12.47
12.39
8.36
O
O
O
O
O
O
O
P
HO
OH
BRUKER
NAME Aug10-2015-OB6081
EXPNO 11
PROCNO 1
Date_ 20150810
Time 11.26
INSTRUM AVIII400
PROBHD 5 mm PABBO BB-
PULPROG zgpg30
TD 65536
SOLVENT DMSO
NS 64
DS 4
SWH 96153.844 Hz
FIDRES 1.467191 Hz
AQ 0.3408372 sec
RG 2050
DW 5.200 usec
DE 6.50 usec
TE 295.2 K
D1 2.00000000 sec
D11 0.03000000 sec
TD0 1
======== CHANNEL f1 ========
NUC1 31P
P1 9.40 usec
PL1 0.00 dB
PL1W 23.83780289 W
SFO1 161.9310633 MHz
======== CHANNEL f2 ========
CPDPRG2 waltz16
NUC2 1H
PCPD2 80.00 usec
PL2 -1.00 dB
PL12 15.55 dB
PL13 19.00 dB
PL2W 12.26963711 W
PL12W 0.27153867 W
PL13W 0.12269637 W
SFO2 400.0416002 MHz
SI 32768
SF 161.9391600 MHz
WDW EM
SSB 0
LB 1.00 Hz
GB 0
PC 1.40
200
150
100
50
0
-50
-100
-150
-200
-250
ppm

compound-10 ^{1}HNMR

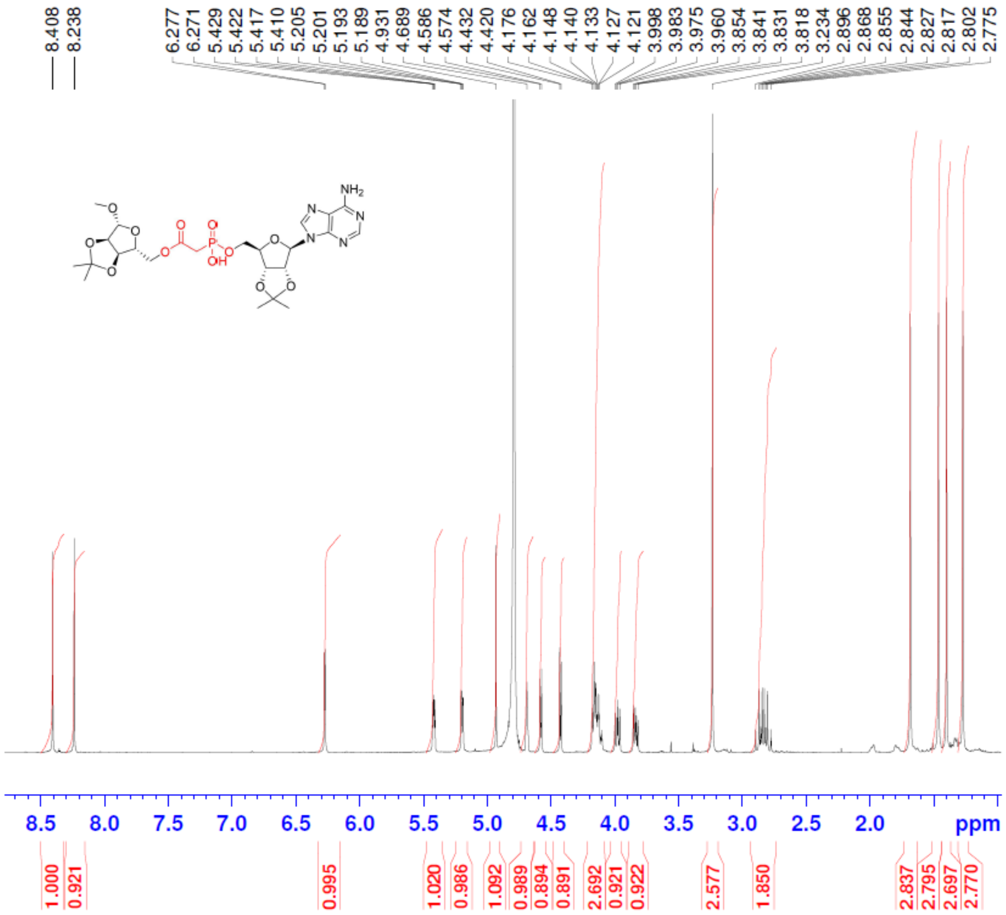

NAME OBB-24
EXPNO 10
PROCNO 1
Date_ 20151021
Time 11.51
INSTRUM spect
PROBHD 5 mm PABBO BB-
PULPROG zg30
TD 65536
SOLVENT D2O
NS 16
DS 2
SWH 10330.578 Hz
FIDRES 0.157632 Hz
AQ 3.1719923 sec
RG 114
DW 48.400 usec
DE 14.00 usec
TE 298.2 K
D1 1.00000000 sec
TD0 1

======== CHANNEL f1 ========
NUC1 1H
P1 10.00 usec
PL1 -0.12 dB
PL1W 19.35150909 W
SFO1 500.1330885 MHz
SI 32768
SF 500.1299569 MHz
WDW EM
SSB 0
LB 0.30 Hz
GB 0
PC 1.00

compound-10 ^{13}CNMR

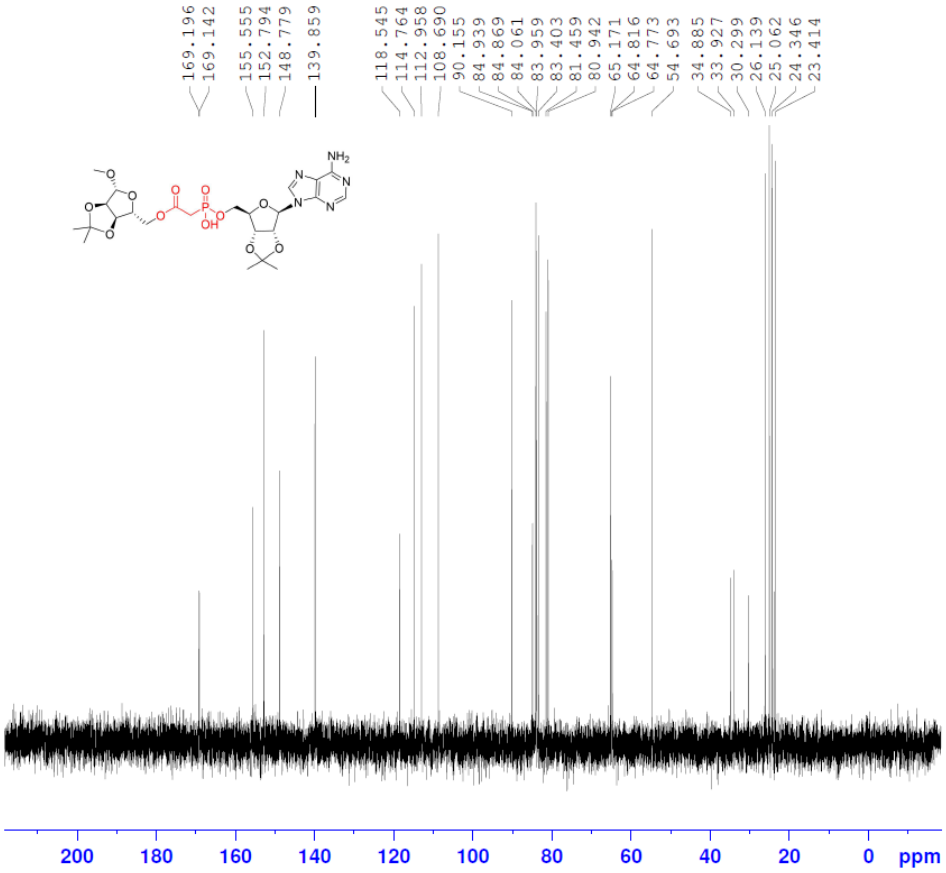

NAME OBB-24
EXPNO 13
PROCNO 1
Date_ 20151021
Time 13.04
INSTRUM spect
PROBHD 5 mm PABBO BB-
PULPROG zgpg30
TD 65536
SOLVENT D2O
NS 2227
DS 4
SWH 29761.904 H:
FIDRES 0.454131 H:
AQ 1.1010548 se
RG 2050
DW 16.800 u:
DE 8.45 u:
TE 298.3 K
D1 2.00000000 se
D11 0.03000000 se
TD0 1

======== CHANNEL f1 =====
NUC1 13C
P1 9.40 u:
PL1 -0.51 dI
PL1W 99.92730713 W
SFO1 125.7703643 MI

======== CHANNEL f2 =====
CPDPRG2 waltz16
NUC2 1H
PCPD2 80.00 u:
PL2 -0.12 dI
PL12 17.94 dI
PL13 21.00 dI
PL2W 19.35150909 W
PL12W 0.30249262 W
PL13W 0.14952536 W
SFO2 500.1320005 MI
SI 32768
SF 125.7577890 MI
WDW EM
SSB 0
LB 1.00 H:
GB 0
PC 1.40

compound-10 ^{31}PNMR

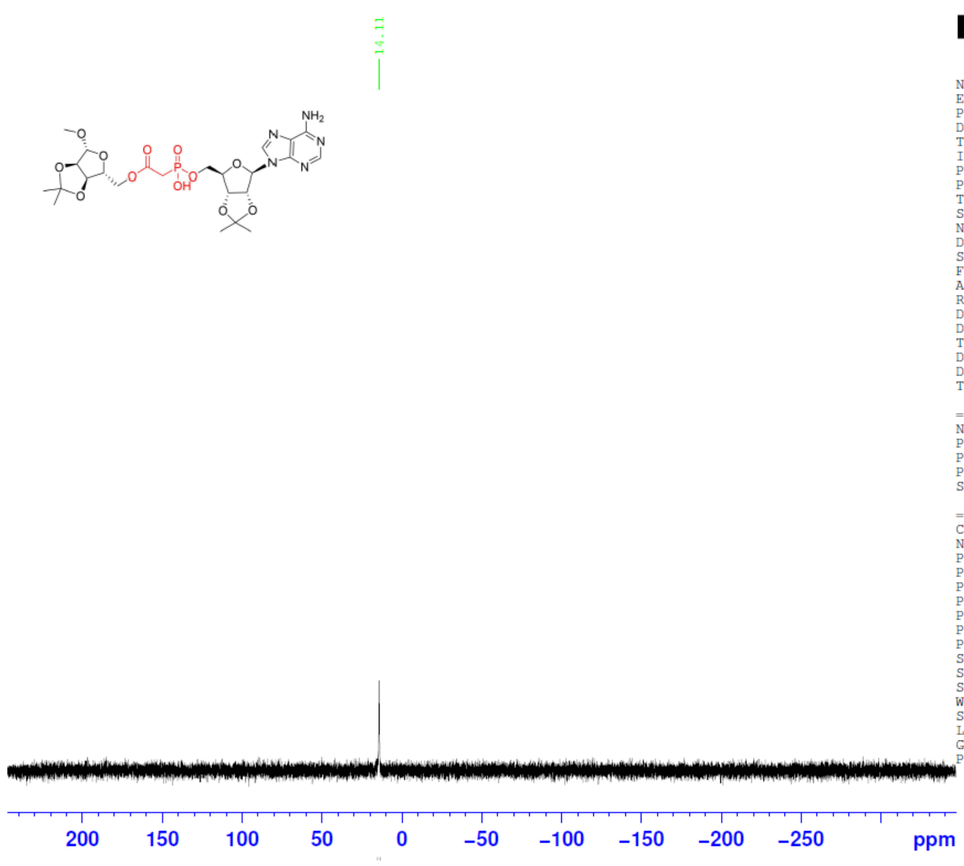


```
NAME        Dec08-2015-OBB24_PUR
EXPNO                 11
PROCNO                 1
Date_           20151208
Time               16.43
INSTRUM         AVIII400
PROBHD   5 mm PABBO BB-
PULPROG          zgpg30
TD                65536
SOLVENT             D2O
NS                   64
DS                    4
SWH           96153.844 Hz
FIDRES         1.467191 Hz
AQ            0.3408372 sec
RG                 2050
DW                5.200 usec
DE                 6.50 usec
TE                298.2 K
D1           2.00000000 sec
D11          0.03000000 sec
TD0                   1

======== CHANNEL f1 ========
NUC1                 31P
P1                  9.40 usec
PL1                 0.00 dB
PL1W         23.83780289 W
SFO1         161.9310633 MHz

======== CHANNEL f2 ========
CPDPRG2          waltz16
NUC2                  1H
PCPD2              80.00 usec
PL2                -1.00 dB
PL12               15.55 dB
PL13               19.00 dB
PL2W         12.26963711 W
PL12W         0.27153867 W
PL13W         0.12269637 W
SFO2         400.0416002 MHz
SI                 32768
SF           161.9391600 MHz
WDW                   EM
SSB                    0
LB                  1.00 Hz
GB                     0
PC                  1.40
```

compound-2 ^{1}HNMR (DOSY)

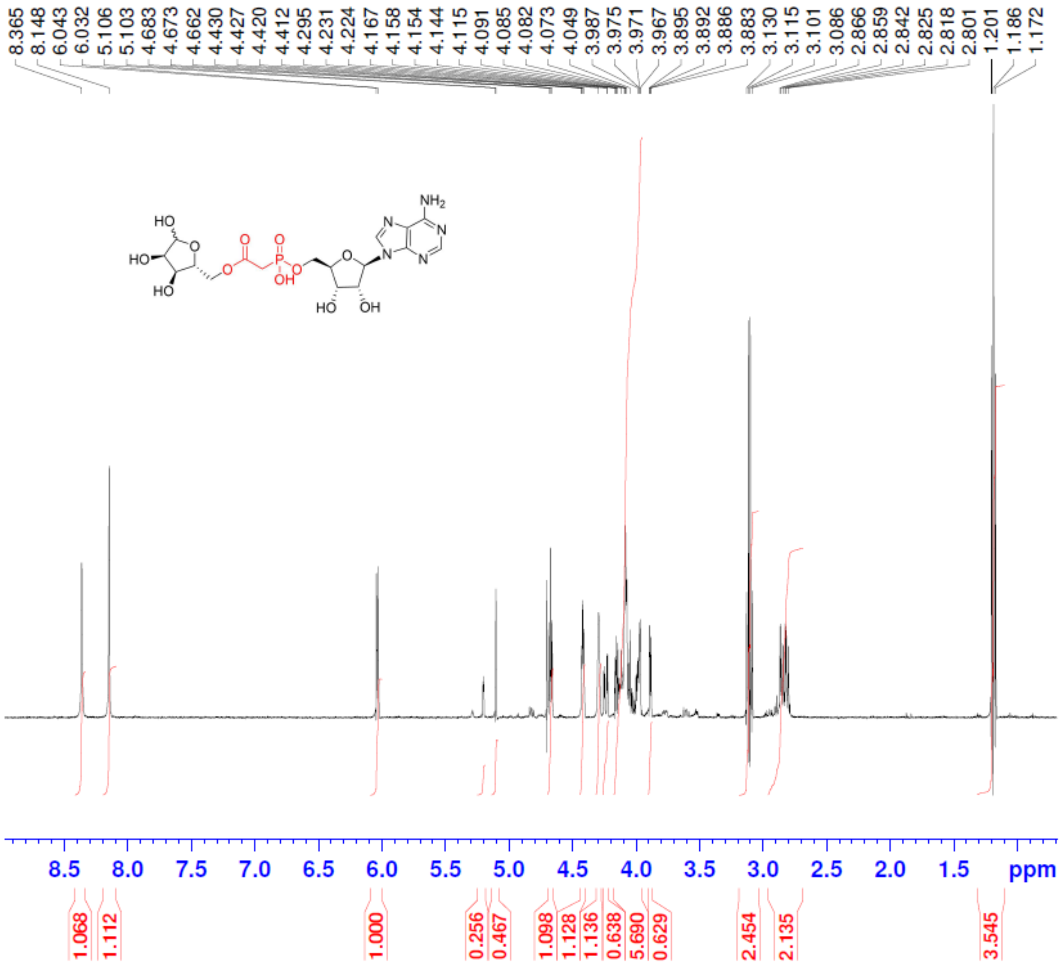


```
NAME                OBB46
EXPNO                  50
PROCNO                  1
Date_            20151218
Time                11.50
INSTRUM             spect
PROBHD    5 mm PABBO BB-
PULPROG      ledbpgp2s1d
TD                  65536
SOLVENT               D2O
NS                     16
DS                      2
SWH             10330.578 Hz
FIDRES           0.157632 Hz
AQ              3.1719923 sec
RG                   22.6
DW                 48.400 usec
DE                   9.73 usec
TE                  298.2 K
D1             1.00000000 sec
D16            0.00020000 sec
D20            0.05000000 sec
D21            0.00500000 sec
TD0                     1

======== CHANNEL f1 ========
NUC1                   1H
P1                  10.00 usec
P2                  20.00 usec
PL1                 -0.12 dB
PL1W          19.35150909 W
SFO1          500.1330885 MHz

====== GRADIENT CHANNEL =====
GPNAM6               sine.100
GPNAM7               sine.100
```

compound-2 ^{13}CNMR

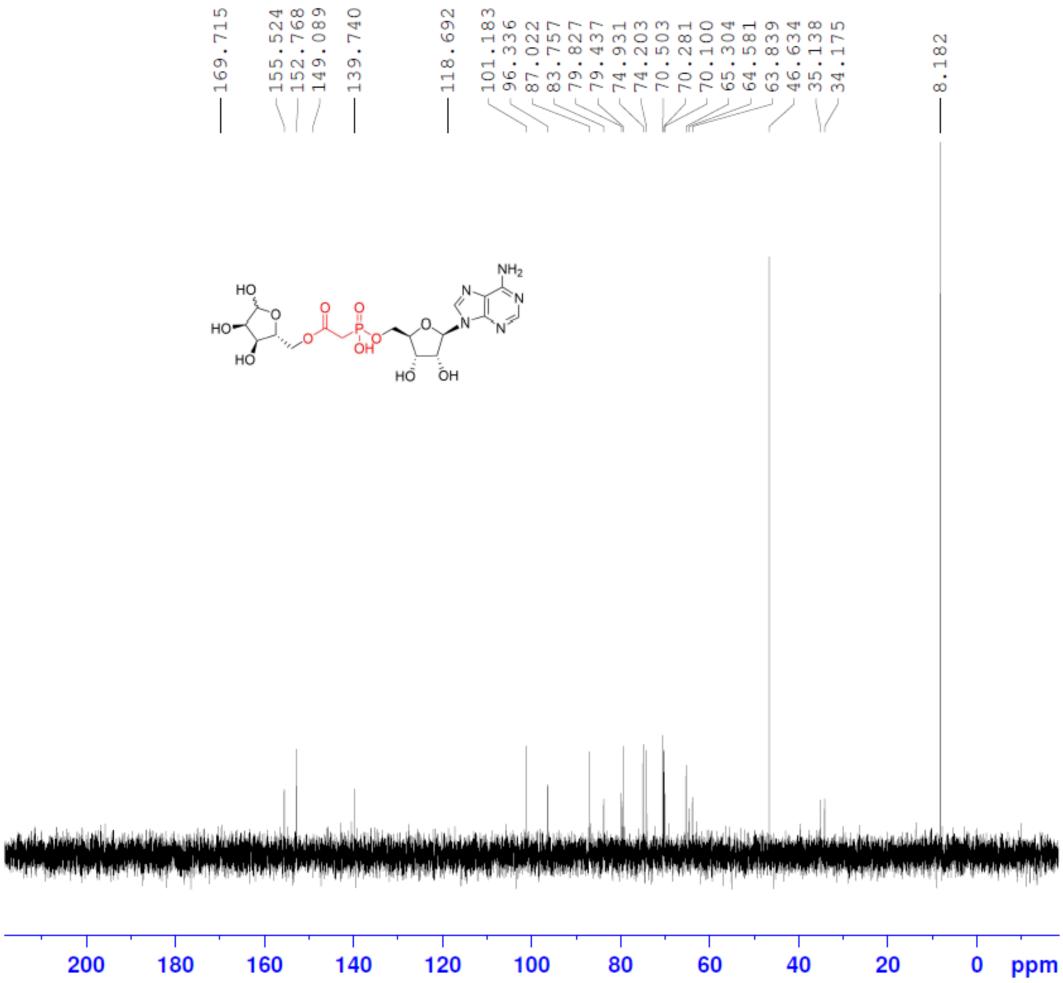


```
NAME            OBB46
EXPNO              22
PROCNO              1
Date_        20151218
Time             9.41
INSTRUM         spect
PROBHD   5 mm PABBO BB-
PULPROG       zgpg30
TD              65536
SOLVENT           D2O
NS                512
DS                  4
SWH         29761.904 Hz
FIDRES       0.454131 Hz
AQ          1.1010548 sec
RG               2050
DW             16.800 usec
DE               8.45 usec
TE              298.2 K
D1         2.00000000 sec
D11        0.03000000 sec
TD0                 1

======== CHANNEL f1 ========
NUC1              13C
P1               9.40 usec
PL1             -0.51 dB
PL1W      99.92730713 W
SFO1      125.7703643 MHz

======== CHANNEL f2 ========
CPDPRG2       waltz16
NUC2               1H
PCPD2           80.00 usec
PL2             -0.12 dB
PL12            17.94 dB
PL13            21.00 dB
PL2W      19.35150909 W
PL12W      0.30249262 W
PL13W      0.14952536 W
SFO2      500.1320005 MHz
SI              32768
SF        125.7577890 MHz
WDW                EM
SSB                 0
LB               1.00 Hz
GB                  0
PC               1.40
```

compound-2 ^{31}PNMR

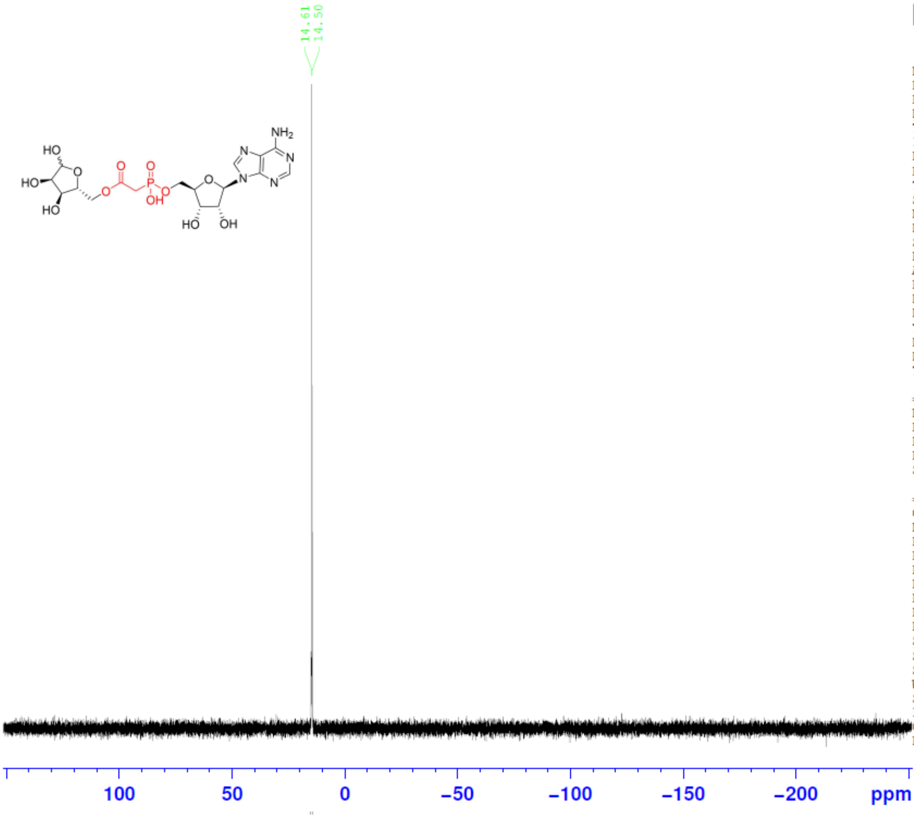

BRUKER

```
NAME            OBB46
EXPNO              24
PROCNO              1
Date_        20151218
Time            10.09
INSTRUM         spect
PROBHD   5 mm PABBO BB-
PULPROG       zgpg30
TD              65536
SOLVENT           D2O
NS                256
DS                  4
SWH         81521.742 Hz
FIDRES       1.243923 Hz
AQ          0.4020041 sec
RG               2050
DW              6.133 usec
DE               6.50 usec
TE              298.2 K
D1         2.00000000 sec
D11        0.03000000 sec
TD0                 1

======== CHANNEL f1 ========
NUC1              31P
P1              10.50 usec
PL1              0.00 dB
PL1W      81.20777893 W
SFO1      202.4462121 MHz

======== CHANNEL f2 ========
CPDPRG2       waltz16
NUC2               1H
PCPD2           80.00 usec
PL2             -0.12 dB
PL12            17.94 dB
PL13            21.00 dB
PL2W      19.35150909 W
PL12W      0.30249262 W
PL13W      0.14952536 W
SFO2      500.1320005 MHz
SI              32768
SF        202.4563350 MHz
WDW                EM
SSB                 0
LB               1.00 Hz
GB                  0
PC               1.40
```

compound-2 HPLC

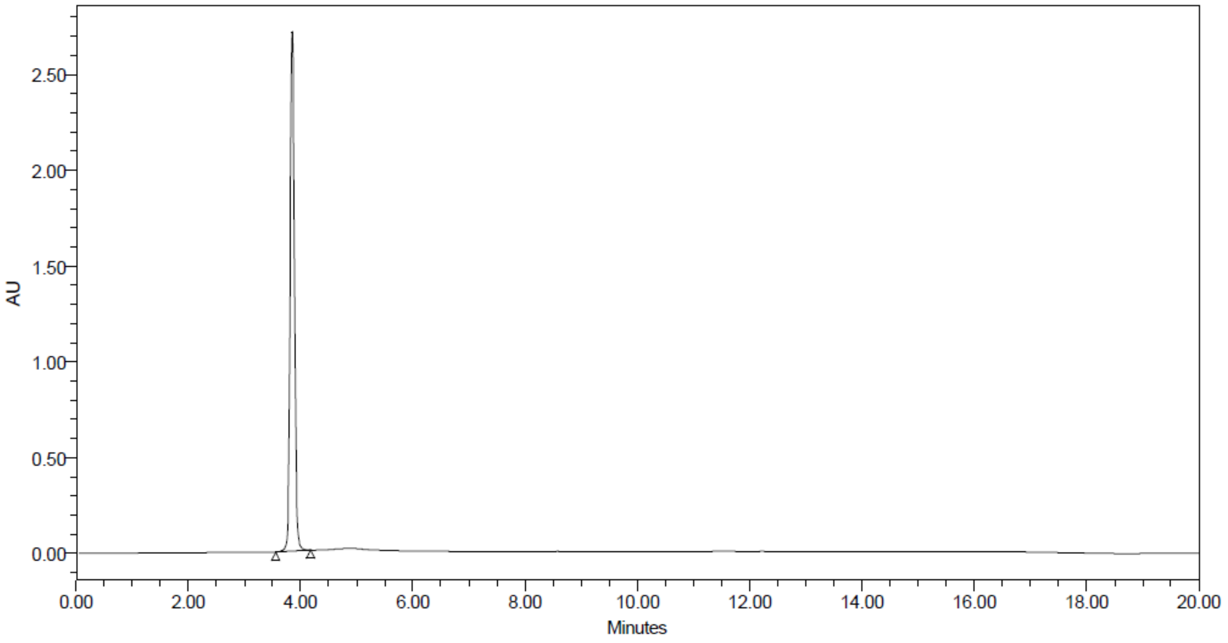

	Peak Name	RT	Area	% Area	Height
1	compound-2	3.854	14751722	100.00	2724648

Confirmation of Expected Formula

Sample-ID	compound-2	Submitter	Ondrej Baszczynski
Analysis Name	po_ob_obb45_345635_44_01_50211.d	Supervisor	Barry Potter
Method used	Confirm Formula Negative 50to1500 loop inj.m	Acquisition Date	26/11/2015 10:36:59
Ionisation Mode	negative electrospray (ESI)		

-MS, 1.0-1.3min #(32-40), -Spectral Bkgrnd

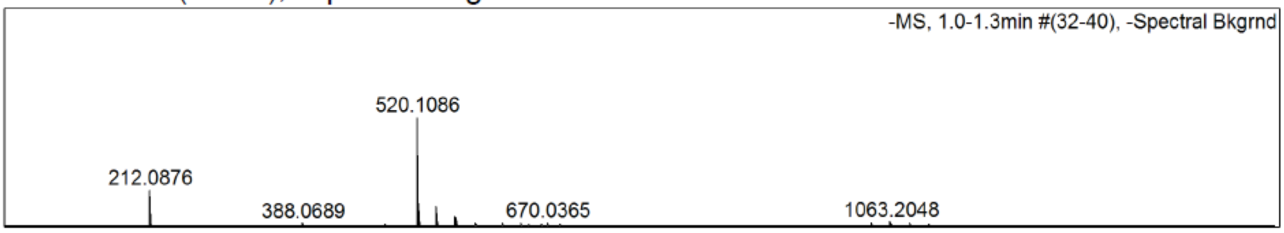

#	m/z	I	I %	Area	S/N
1	212.0876	3329	33.3	42	71219.7
2	520.1086	10006	100.0	790	16233.1
3	521.1114	2135	21.3	164	3467.2
4	522.1139	446	4.5	32	725.4
5	542.0918	1811	18.1	155	3018.5
6	543.0931	359	3.6	30	599.9
7	564.0744	837	8.4	73	1434.8
8	670.0365	314	3.1	29	1168.4
9	1063.2048	361	3.6	49	4160.5
10	1085.1838	266	2.7	35	3488.5

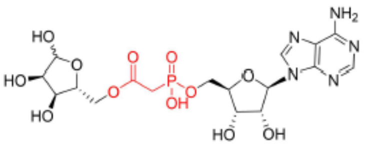

Generate Molecular Formula Parameters

Charge	Tolerance	SearchRadius	H/C Ratio min.	H/C Ratio max.	Electron Conf.	Nitrogen Rule	sigma limit
negative	10 ppm	0.05 m/z	0	3	both	true	0.05

Expected Formula C17 H24 N5 O12 P **Adduct(s):** H, Na

#	meas. m/z	theo. m/z	Err[ppm]	Sigma	Formula
1	520.1086	520.108083	0.20	0.0042	C 17 H 23 N 5 O 12 P 1
